# Supplementary material for: Identification of Freezing-Responsive microRNAs and Their Targets in Chinese Jujube by Small RNA and Degradome Sequencing
Source: Int J Mol Sci. 2026 Sep 4;27(17):7912. doi: 10.3390/ijms27177912 (PMC13566890; doi:10.3390/ijms27177912)
Supplement: Supplementary file 1 [file ijms-27-07912-s001.zip › Supplementary Material S2/novel_miR_176.pdf]

Provisional ID	: novel_miR_176
Score total	: 24769.7
Score for star read(s)	: 3.9
Score for read counts	: 24767.9
Score for mfe	: 0
Score for randfold	: -2.2
Score for cons. seed	:
Total read count	: 48593
Mature read count	: 48582
Loop read count	: 3
Star read count	: 8

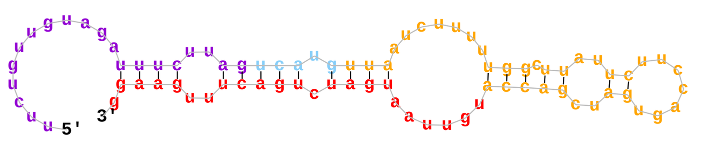

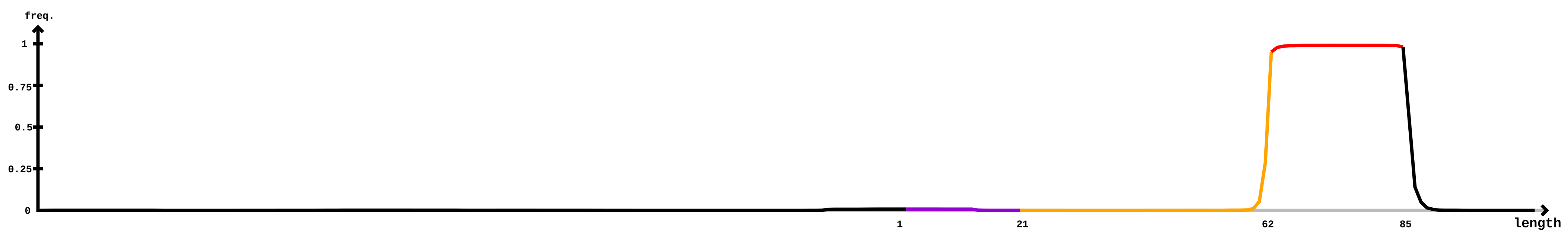

Star Mature

```
5'- uucgcgcagccccuguccaauucuaguuucaauuuuuauugugucacaauuaauucgaacggaacaacguggcaacuguucaauucugauuucaauuuuuuuuguuacauaauuaguucaaauaaaacaauuugacuacuguuuucuguuguagauuucuuagucauguuaaucuuuuuggcuuauucuuccagugaucgaccauguuaaugaucugacuuugaaggcuauuuagggcacuuaaauauau -3' obs
    uucgcgcagccccuguccaauucuaguuucaauuuuuauugugucacaauuaauucgaacggaacaacguggcaacuguucaauucugauuucaauuuuuuuuguuacauaauuaguucaaauaaaacaauuugacuacuguuuucuguuguagauuucuuagucauguuaaucuuuuuggcuuauucuuccagugaucgaccauguuaaugaucugacuuugaaggcuauuuagggcacuuaaauauau     exp
    ........((((.......................((((((.((((..((((.((((((((.(((((....)).)))...))))).))).))))....)))).)))))))((((.(((((......)))))))))..((((((.....((((((..((((.((((((.(((...((((........)))).))).)).)))))))).))))))).....))))))......))))............
```

read	reads	mm	sample
uucgcgcagccccugucc	2	0	LT-1
uucgcgcagccccugucc	1	0	LCK-3
uucgcgcagccccugucA	1	1	LT-1
uucgcgcagccccugucc	1	0	LT-2
uucgcgcagccccugucA	2	1	JCK-2
uucgcgcagccccugucc	1	0	JCK-3
uucgcgcagccccugucU	2	1	JCK-2
uucgcgcagccccugucca	5	0	JT-3
uucgcgcagccccugucca	4	0	JT-2
uucgcgcagccccugucca	1	0	LT-3
.ucgcgcagccccuguccC	2	1	LT-2
.ucgcgcagccccuguccaauu	1	0	LT-1
caauucuaguuucaauuuuuuauu	1	0	LT-1
uuuuuauugugucacaauuaauucgaacgU	1	1	JCK-2
uuuuuauugugucacaauuaauucgaacgU	1	1	JCK-3
uugugucacaauuaauucgaacg	7	0	LCK-3
ucacaauuaauucgaacggaacaacgu	3	0	JCK-2
acaauuaauucgaacggaacaacguggc	5	0	JCK-1
Uaauuaauucgaacggaacaacg	7	1	LCK-3
auuaauucgaacggaacaacgugg	1	0	LT-3
uuaauucgaacggaacaacg	3	0	JT-2
uuaauucgaacggaacaacg	8	0	JT-3
uuaauucgaacggaacaacgug	2	0	LT-1
cgaacggaacaacguggcaacuguuca	2	0	LCK-3
aacggaacaacguggcaacuguuc	1	0	LT-1
cggaacaacguggcaacuguucaauu	1	0	LT-1
caacguggcaacuguucaauucugauuuc	1	0	LT-2
aacguggcaacuguucaauucugauuuca	1	0	LT-3
cguggcaacuguucaauucuga	1	0	LT-1
gcaacuguucaauucugauu	1	0	JCK-1
gcaacuguucaauucugauu	1	0	JCK-3
aacuguucaauucugauuuc	5	0	LT-3
cuguucaauucugauuuca	1	0	LT-3

Star Mature

uucgcgcagccccuguccaauucuaguuucaauuuuuauuguguccacaauuaauucgaacggaacaacguggcaacuguucaauucugauuucaauuuuuuuguuacauaauuaguucaaauaaaacaauuugacuacuguuuucuguuguagauuucuuagucauguuaaucuuuuuggcuuauucuuccagugaucgaccauguuaaugaucugacuuugaaggcuauuuagggcacuuaaauauau

cuguucaauucugauuucaauuu	3	0	LT-1
cuguucaauucugauuucaauuuuC	1	1	LT-1
aNaauuugacuacuguuuucug	1	1	JCK-1
caauuugacuacuguuuucugu	3	0	JCK-3
caauuugacuacuguuuucugu	4	0	JCK-2
caauuugacuacuguuuucugu	2	0	JCK-1
auuugacuacuguuuucug	1	0	LCK-3
auuugacuacuguuuucug	1	0	LT-3
auuugacuacuguuuucuguu	3	0	LCK-3
auuugacuacuguuuucuguuguaga	4	0	LCK-1
auuugacuacuguuuucuguuguaga	2	0	LT-1
auuugacuacuguuuucuguuguaga	1	0	JCK-2
auuugacuacuguuuucuguuguaga	4	0	LT-2
auuugacuacuguuuucuguuguaga	2	0	LCK-3
auuugacuacuguuuucuguuguaga	3	0	JCK-1
auuugacuacuguuuucuguuguaga	5	0	LCK-2
uuugacuacuguuuucuguu	2	0	LT-2
uuugacuacuguuuucuguuguaga	25	0	LT-1
uuugacuacuguuuucuguuguaga	72	0	LCK-3
uuugCcuacuguuuucuguuguaga	1	1	LT-2
uuugacuacuguuuucuguuguaga	29	0	LT-2
uuugacuacuguuuucuguuguaga	2	0	JCK-2
uuugacuacuguuuucuguuguaga	2	0	JCK-3
uuugacuacuguuuucuAuuguaga	1	1	LCK-1
uuugacuCcuguuuucuguuguaga	1	1	LT-2
uuugaGuacuguuuucuguuguaga	1	1	LT-2
uuugacuacuguuuucuguuguaga	38	0	LCK-1
uuugacuacuguuuucuguuguaga	1	0	JCK-1
uuugacuacuguuuucuguuguagC	1	1	LCK-3
uuugacuacuguuuucuguuguCga	1	1	LT-2
uuugacuCcuguuuucuguuguaga	1	1	LCK-3
uuugacuacuguuuucuguuguaga	12	0	LT-3
uuugacuacuguuuucuguuguaga	43	0	LCK-2
uuugacuacuguuuucuguuguagau	8	0	LCK-2
uuugacuacuguuuucuguuguagau	10	0	LCK-1
uuugacuacuguuuucuguuguagau	1	0	JCK-3
uuugacuacuguuuucuguuguagau	3	0	JCK-2
uuugacuacuguuuucuguuguagau	1	0	JCK-1
uugacuacuguuuucuguuguaga	2	0	LT-3
uugacuacuguuuucuguuguaga	8	0	LT-1
uugacuacuguuuucuguuguaga	15	0	LT-2
uugacuacuguuuucuguuguaga	1	0	JCK-2
Cugacuacuguuuucuguuguaga	1	1	LT-2
uugacuacuguuuucuguuguaga	4	0	LCK-3
uugacuacuguuuucuguuguaga	1	0	JCK-3
uugacuCcuguuuucuguuguaga	2	1	LT-2
uugacuacuguuuucuguuguagaA	2	1	LCK-3
uugacuacuguuuucuguuguagau	3	0	LT-2
uugacuacuguuuucuguuguagauu	1	0	LT-1
ugacuacuguuuucuguuguaga	1	0	JCK-1
ugacuacuguuuucuguuguaga	1	0	JCK-2
uacuguuuucuguuguaga	4	0	LT-3
uacuguuuucuguuguaga	5	0	LT-1
uacuguuuucuguuguagau	1	0	JCK-2
uacuguuuucuguuguagau	1	0	JCK-3
uacuguuuucuguuguagauu	4	0	LT-2
acuguuuucuguuguaga	2	0	LT-2
acuguuuucuguuguaga	3	0	LCK-3
acuguuuucuguuguaga	1	0	LT-3
acuguuuucuguuguaga	1	0	LT-1
acuguuuucuguuguagau	1	0	JCK-1
acuguuuucuguuguagau	3	0	JCK-2
acuguuuucuguuguagauu	1	0	LT-1
acuguuuucuguuguagauuucuuag	1	0	JCK-3
cuguuuucuguuguagau	1	0	JCK-2
cuguuuucuguuguagau	1	0	LT-1
cuguuuucuguuguagauu	5	0	LT-2
cuguuuucuguuguagauuucuua	2	0	JCK-2
uguuuucuguuguagauuucuuagu	2	0	JCK-3
uguuuucuguuguagauuucuuagu	1	0	LT-2

Star · Mature

uucgcgcagccccuguccaauucuaguuucaauuuuuauugugucacaauuaauucgaacggaacaacguggcaacuguucaauucugauuucaauuuuuuuuguuacauaauuaguucaaauaaaacaauuugacuacuguuuucuguuguagauuucuuagucauguuaaucuuuuuggcuuauucuuccagugaucgaccauguuaaugaucugacuuugaaggcuauuuagggcacuuaaauauau

uguuuucuguuguagauuucuuagu	1	0	JCK-1
guuuucuguuguagauuucuuagu	1	0	LT-1
uuuucuguuguagauuucuuagucaug	1	0	LCK-3
uuuucuguuguagauuucuuagucauguu	1	0	JCK-2
uuucuguuguagauuucuuagucauguu	1	0	LT-3
uucuguuguagauuucuuag	3	0	LT-2
ucuguuguagauuucuuagucauguu	2	0	LT-2
uguuaaucuuuuuggcuuauucuuccag	1	0	LT-1
uuGggcuuauucuuccaguga	1	1	LT-1
auucuuccagugaucgaccaug	1	0	LT-2
uccagugaucgaccauguuaaugaucu	2	0	JCK-3
uccagugaucgaccauguuaaugaucu	1	0	JCK-2
uccagugaucgaccauguuaaugaucu	2	0	JCK-1
aucgaccauguuCaugaucugacuuugaa	1	1	LT-2
ucgaccauguuaaugaucugacu	2	0	JCK-3
ucgaccauguuaaugaucugacu	1	0	LT-1
uAgaccauguuaaugaucugacuuugaagg	1	1	JCK-2
ucgaccauguuaaugaucugacuuugaagg	8	0	LCK-2
ucgaccauguuaaugaucugacuuugaagg	3	0	JCK-2
ucgaccauguuaaugaucugacuuugaagg	2	0	LT-1
ucgaccauguuaaugaucugacuuugaagg	1	0	LCK-1
ucgaccauguuaaugaucugacuuugaagg	4	0	LT-2
ucgaccauguuaaugaucugacuuugaagg	1	0	JCK-1
ucgaccauguuaaugaucugacuuugaagg	1	0	LT-3
ucgaccauguuaaugaucugacuuugaagg	7	0	LCK-3
uAgaccauguuaaugaucugacuuugaagg	1	1	JCK-1
uAgaccauguuaaugaucugacuuugaagg	2	1	JCK-3
cgaccauguuaaugaucugacuuugaa	1	0	LT-2
cgaccauguuaaugaucugacuuugaagg	1	0	LCK-3
cgaccauguuaaugaucugacuuugaagg	4	0	LT-2
gaccauguuaaugaucugacuuugaagg	1	0	JCK-2
gaccauguuaaugaucugacuuugaagg	3	0	LCK-3
gaccauguuaaugaucugacuuugaagg	1	0	JCK-1
gaccauguuaaugaucugacuuugaaggc	1	0	LT-1
accauguuaaugaucugacuuugaag	3	0	JCK-2
accauguuaaugaucugacuuugaag	1	0	LT-3
accauguuaaugaucugacuuugaagg	2	0	LT-1
accauguuaaugaucugacuuugaagg	1	0	JT-1
accauguuaaugaucugacuuugaagg	1	0	JCK-3
accauguuaaugaucugacuuugaagg	14	0	LCK-3
accauguuaaugaucugacuuugaagg	5	0	LT-3
accauguuaaugaucugacuuugaagg	4	0	JCK-2
accauguuaaugaucugacuuugaaggc	1	0	JCK-1
accauguuaaugaucugacuuugaaggc	5	0	LCK-3
accauguuaaugaucugacuuugaaggc	4	0	LT-2
accauguuaaugaucugacuuugaaggc	7	0	JCK-3
accauguuaaugauNugacuuugaaggc	1	1	LCK-1
accauguuaaugaucugacuuugaaggc	11	0	LCK-1
accauguuaaugaucugacuuugaaggc	5	0	JCK-2
accauguuaaugaucugacuuugaaggc	4	0	LT-1
accauguuaaugaucugacuuugaaggc	7	0	LCK-2
accauAuuaaugaucugacuuugaaggc	1	1	JCK-3
accauguuaaugaucugacuuugaaggA	1	1	LT-2
accauguuaaugaucugacuuugaaggcu	1	0	JCK-2
ccauguuaaugCucugacuuuga	1	1	JCK-1
ccauguuaaugaucugacuuuga	5	0	JCK-1
ccauguuaaugaucugacuuuga	2	0	JCK-3
ccauguuaaugaucugacuuuga	2	0	LT-1
ccauguuaaugaucugacuuugaa	2	0	JCK-2
ccauguuaaugaucugacuuugaa	1	0	JCK-3
ccauguuaaugaucugacuuugaag	4	0	LT-1
ccauguuaaugaucugacuuugaag	1	0	LT-3
ccauguuaaugaucugacuuugaag	3	0	LT-2
ccauguuaaugaucugacuuugaag	4	0	LCK-3
ccauguuaaugaucugacuuugaagg	31	0	LCK-2
ccauguuaaugaucugacuuugaagg	7	0	JT-1
ccauguuaaugaucugacuuugaagg	25	0	LT-2
ccauguuaaugaucugacuuugaagg	7	0	LT-3
ccauguuaaugaucugacuuugaagg	8	0	LT-1
ccauguuaaugaucugacuuugaagg	4	0	JCK-3

Star Mature

```
uucgcgcagccccuguccaauucuaguuucaauuuuuauugugucacaauuaauucgaacggaacaacguggcaacuguucaauucugauuucaauuuuuuuuguuacauaauuaguucaaauaaaacaauuugacuacuguuuucuguuguagauuucuuagucauguuaaucuuuuuggcuuauucuuccagugaucgaccauguuaaugaucugacuuugaaggcuauuuagggcacuuaaauauau
.........................................................................................................................................................................................................ccauguuaaugaucugacuuugaagg.......................   27   0   LCK-1
.........................................................................................................................................................................................................ccauguuaaugaucugacuuugaagg.......................   3    0   JCK-2
.........................................................................................................................................................................................................ccauguuaaugaucugacuuugaagg.......................   39   0   LCK-3
.........................................................................................................................................................................................................ccauguuaaugaucugacuuugaaggA......................   1    1   LT-3
.........................................................................................................................................................................................................ccauguuCaugaucugacuuugaaggc......................   2    1   JCK-2
.........................................................................................................................................................................................................ccauguuaaugaucugacuuugaaggc......................   41   0   JCK-2
.........................................................................................................................................................................................................ccauguuaaugaucugacuuugaaggc......................   11   0   LT-3
.........................................................................................................................................................................................................ccauguuaaugaucugacuuugaaggc......................   25   0   JCK-1
.........................................................................................................................................................................................................ccauguuaaugaucugacuuugaaggA......................   2    1   JCK-2
.........................................................................................................................................................................................................ccauguuaaugaucugacuuugaaggc......................   10   0   LCK-3
.........................................................................................................................................................................................................ccauguuCaugaucugacuuugaaggc......................   1    1   LT-2
.........................................................................................................................................................................................................ccauguuaaugaucugacuuugaaggA......................   9    1   LCK-2
.........................................................................................................................................................................................................ccCuguuaaugaucugacuuugaaggc......................   1    1   LT-3
.........................................................................................................................................................................................................ccauguuCaugaucugacuuugaaggc......................   1    1   JCK-3
.........................................................................................................................................................................................................ccauguuaaugaucugacuuugaaggc......................   12   0   LT-2
.........................................................................................................................................................................................................ccauguuaaugaucugacuuugaaggA......................   3    1   LCK-1
.........................................................................................................................................................................................................ccauguuaaugaucugacuuugaaggc......................   5    0   LT-1
.........................................................................................................................................................................................................ccauguuaaugaucugacuuuUaaggc......................   1    1   JCK-2
.........................................................................................................................................................................................................ccauguuaaugaucugacuuugaaggc......................   30   0   JCK-3
.........................................................................................................................................................................................................ccauguuaaugaucugacuuugaaggcu.....................   5    0   LCK-3
.........................................................................................................................................................................................................ccauguuaaugaucugacuuugaaggcu.....................   5    0   JCK-2
.........................................................................................................................................................................................................ccauguuaaugaucugacuuugaaggcu.....................   8    0   JCK-3
.........................................................................................................................................................................................................ccauguuaaugaucugacuuugaaggcu.....................   1    0   LT-3
.........................................................................................................................................................................................................ccauguuaaugaucugacuuugaaggcu.....................   8    0   LT-2
.........................................................................................................................................................................................................ccauguuaaugaucugacuuugaaggcu.....................   4    0   JCK-1
.........................................................................................................................................................................................................ccauguuaaugaucugacuuugaaggcua....................   3    0   LT-1
.........................................................................................................................................................................................................ccauguuaaugaucugacuuugaaggcua....................   1    0   LT-3
.........................................................................................................................................................................................................ccauguuaaugaucugacuuugaaggcua....................   3    0   JCK-2
.........................................................................................................................................................................................................ccauguuaaugaucugacuuugaaggcua....................   2    0   LT-2
.........................................................................................................................................................................................................ccauguuaaugaucugacuuugaaggcua....................   2    0   JCK-1
.........................................................................................................................................................................................................ccauguuaaugaucugacuuugaaggcua....................   6    0   LCK-3
.........................................................................................................................................................................................................ccauguuaaugaucugacuuugaaggcua....................   2    0   JCK-3
.........................................................................................................................................................................................................ccauguuaaugaucugacuuugaaggcuau...................   2    0   LT-2
.........................................................................................................................................................................................................ccauguuaaugaucugacuuugaaggcuau...................   2    0   LT-1
.........................................................................................................................................................................................................ccauguuaaugaucugacuuugaaggcuau...................   4    0   LCK-3
..........................................................................................................................................................................................................cauguuaaugaucugacuuuga..........................   1    0   LT-3
..........................................................................................................................................................................................................cauguuaaugaucugacuuugaa.........................   1    0   LCK-3
..........................................................................................................................................................................................................cauguuaaugaucugacuuugaa.........................   1    0   LT-3
..........................................................................................................................................................................................................cauguuaaugaucugacuuugaag........................   8    0   LT-2
..........................................................................................................................................................................................................cauguuaaugaucugacuuugaag........................   7    0   JT-2
..........................................................................................................................................................................................................cauguuaaugaucugacuuugaag........................   7    0   LT-1
..........................................................................................................................................................................................................cauguuaaugaucugacuuugaag........................   2    0   LT-3
..........................................................................................................................................................................................................cauguuaaugaucugacuuugaag........................   5    0   JT-3
..........................................................................................................................................................................................................cauguuaaugaucugacuuugaag........................   6    0   LCK-3
..........................................................................................................................................................................................................cauguuaaugaucugacuuugaag........................   4    0   JCK-2
..........................................................................................................................................................................................................cauguuaaugaucugacuuugaag........................   1    0   JCK-3
..........................................................................................................................................................................................................cauguuaauAaucugacuuugaagg.......................   1    1   LT-3
..........................................................................................................................................................................................................cauguuaaugaucuNacuuugaagg.......................   1    1   LCK-1
..........................................................................................................................................................................................................cauguuaaugaucugCcuuugaagg.......................   1    1   LT-2
..........................................................................................................................................................................................................cauguuaaugaucugacuuugaagg.......................   68   0   LT-3
..........................................................................................................................................................................................................cauguuaaugaucugacuuugaagg.......................   51   0   JCK-1
..........................................................................................................................................................................................................cauguuaaugaucugacuuugaagg.......................   242  0   LCK-3
..........................................................................................................................................................................................................cauAuuaaugaucugacuuugaagg.......................   1    1   LCK-2
..........................................................................................................................................................................................................cauguuaaugaucugacuuugNagg.......................   1    1   LT-2
..........................................................................................................................................................................................................cauguuaaugaucugacuuugGagg.......................   1    1   LT-2
..........................................................................................................................................................................................................cauguuaaugaucugacuuugaagg.......................   30   0   JCK-3
..........................................................................................................................................................................................................cNuguuaaugaucugacuuugaagg.......................   1    1   JT-1
..........................................................................................................................................................................................................cCuguuaaugaucugacuuugaagg.......................   3    1   LT-2
..........................................................................................................................................................................................................cauguuaaugaucugacuuugaagg.......................   49   0   JCK-2
..........................................................................................................................................................................................................cauguuCaugaucugacuuugaagg.......................   1    1   LCK-1
..........................................................................................................................................................................................................cauguuaGugaucugacuuugaagg.......................   1    1   LCK-1
..........................................................................................................................................................................................................cauguuaaugaucugacuuugaagg.......................   79   0   LT-1
..........................................................................................................................................................................................................cauguuaaugaucugacuuugaagg.......................   28   0   JT-1
..........................................................................................................................................................................................................cauguuaaugaucugacuuugaagg.......................   7    0   JT-2
..........................................................................................................................................................................................................cauguuCaugaucugacuuugaagg.......................   1    1   LCK-2
..........................................................................................................................................................................................................cauguuaCugaucugacuuugaagg.......................   1    1   LCK-2
..........................................................................................................................................................................................................cauguuaaugaucugacuuugaagg.......................   156  0   LT-2
..........................................................................................................................................................................................................cauguuaaugaucNgacuuugaagg.......................   1    1   LCK-1
..........................................................................................................................................................................................................cauguuaaugaucuAacuuugaagg.......................   1    1   LT-3
..........................................................................................................................................................................................................cauguuaCugaucugacuuugaagg.......................   1    1   LT-2
```

Star Mature

uucgcgcagccccuguccaauucuaguuucaauuuuuauugugucacaauuaauucgaacggaacaacguggcaacuguucaauucugauuucaauuuuuuuuguuacauaauuaguucaaauaaaacaauuugacuacuguuuucuguuguagauuucuuagucauguuaaucuuuuuggcuuauucuuccagugaucgaccauguuaaugaucugacuuugaaggcuauuuagggcacuuaaauauau

cauguuCaugaucugacuuugaagg	1	1	LCK-3
cauguuCaugaucugacuuugaagg	1	1	LT-3
cauguuaaugaucugacuuugaagg	9	0	JT-3
caNguuaaugaucugacuuugaagg	1	1	LT-2
cauguuaaugaucugacuuugaagg	203	0	LCK-2
cauguuaaugaucugacuuugaagg	204	0	LCK-1
cauguuCaugaucugacuuugaaggc	3	1	LT-2
cauguuaaugaucugacuuugaaggc	2	0	JT-2
cauguuaaugaucugacuuugaaggc	27	0	JT-1
cauguuaaugaucugacuuugaaggA	18	1	LCK-2
cauguuaaugaucugacCuugaaggc	1	1	LCK-2
cauguuaaugaucugacuuugaaggA	2	1	JCK-1
cCuguuaaugaucugacuuugaaggc	1	1	LT-2
cauguuCaugaucugacuuugaaggc	1	1	LCK-2
cauguuaaugaucugacuuugaaggc	57	0	JCK-1
cauguuaaugaucugacuuugaaggc	114	0	JCK-2
cauguuaaugaucugacuuugaaggA	2	1	JT-3
cauguuaaugaucugacuuugaaggA	8	1	LCK-3
caugCuaaugaucugacuuugaaggc	1	1	LT-2
cauguuaaugaucugacuuugaaggA	3	1	JT-2
cauguuaCugaucugacuuugaaggc	1	1	LT-2
cauguuaaugaucugacuuugaaggA	7	1	LT-2
cauguuaaugaucugacuuugaaggc	110	0	LCK-2
cauguuaaugaucugacuuugaaggG	2	1	LCK-3
cauguuaaugaucuNacuuugaaggc	1	1	LCK-1
cauguuaaugaucugacuuugaaggc	47	0	JCK-3
cauguuaaugaucugacuuugaaggA	5	1	LT-1
cauguuaaugaucugacuuugaaggc	30	0	LT-3
cauguuaaugCucugacuuugaaggc	1	1	JCK-2
cauguuaaugCucugacuuugaaggc	1	1	LCK-3
cauguuaaugaucugacuuugaaggc	71	0	LT-2
cauguuCaugaucugacuuugaaggc	1	1	JCK-1
cauguuaaugaucugacuuugaaggc	22	0	LT-1
cCuguuaaugaucugacuuugaaggc	1	1	LCK-1
cauguuaaugaucugacuuugaaggc	96	0	LCK-1
cauguuaaugaucugacuuugaaggG	1	1	JT-1
cauguuCaugaucugacuuugaaggc	1	1	LT-3
cauguuaaugaucugacuuugaaggc	114	0	LCK-3
cauguuaaugaucugacuuugaaggc	1	0	JT-3
cauguuCaugaucugacuuugaaggc	1	1	LCK-1
cauguuaCugaucugacuuugaaggc	1	1	JCK-3
cauguuaaugaucugacuuugaaggA	17	1	LCK-1
cauguuaaugaucugacuuugaaggcu	10	0	LT-1
cauguuaaugaucugacuuugaaggcu	5	0	JCK-1
cauguuaaugaucugacuuugaaggcu	12	0	JCK-3
cauguuaaugaucugacuuugaaggcu	11	0	JCK-2
cCuguuaaugaucugacuuugaaggcu	1	1	JCK-2
cauguuaaugaucugacuuugaaggcu	13	0	LT-2
cauguuaaugaucugacuuugaaggcu	8	0	LCK-3
cauguuaaugaucugacuuugaaggcu	5	0	LT-3
cauguuaaugaucugacuuugaaggcua	6	0	LT-3
cauguuaaugaucugacuuugaaggcua	7	0	LT-1
cauguuaaugaucugacuuugaaggcua	9	0	JCK-3
cauguuaaugaucugacuuugaaggcua	12	0	LT-2
cauguuaaugaucugacuuugaaggcua	4	0	JCK-1
cauguuaaugaucugacuuugaaggcua	6	0	JCK-2
cauguuaaugaucugacuuugaaggcua	6	0	LCK-3
cauguuaaugaucugacuuugaaggcuau	1	0	LT-2
cauguuaaugaucugacuuugaaggcuau	2	0	LCK-2
cauguuaaugaucugacuuugaaggcuau	1	0	LT-3
cauguuaaugaucugacuuugaaggcuau	4	0	LT-1
cauguuaaugaucugacuuugaaggcuau	1	0	JCK-3
cauguuaaugaucugacuuugaaggcuau	1	0	JCK-1
cauguuaaugaucugacuuugaaggcuau	1	0	JCK-2
cauguuaaugaucugacuuugaaggcuau	3	0	LCK-1
cauguuaaugaucugacuuugaaggcuauC	1	1	LT-2
cauguuaaugaucugacuuugaaggcuauu	2	0	LT-1
cauguuaaugaucugacuuugaaggcuauu	1	0	JCK-2
cauguuaCugaucugacuuugaaggcuauu	1	1	LT-1
auguuaaugaucugacuuuga	2	0	LT-2

Star Mature

uucgcgcagccccuguccaauucuaguuucaauuuuuauuguguccacaauuaauucgaacggaacaacguggcaacuguucaauucugauuucaauuuuuuuuguuacauaauuaguucaaauaaaacaauuugacuacuguuuucuguuguagauuucuuagucauguuaaucuuuuuggcuuauucuuccagugaucgaccauguuaaugaucugacuuugaaggcuauuuagggcacuuaaauauau

...auguuaaugaucugacuuugaa...	1	0	LT-1
...auguuaaugaucugacuuugaa...	2	0	JCK-3
...auguuaaugaucugacuuugaa...	4	0	JCK-2
...auguuaaugaucugacuuugaa...	4	0	JCK-1
...auguuaaugaucugacuuugaa...	1	0	LT-3
...auguuaaugaucugacuuugaag...	3	0	JCK-1
...auguuaaugaucugacuuugaag...	41	0	LCK-3
...auguuCaugaucugacuuugaag...	1	1	LT-3
...auguuaaugaucugacuuugCag...	1	1	LT-2
...auguuaaugaucugacuuugaag...	15	0	LT-1
...auguuaaugaucugacuuugaag...	10	0	JCK-2
...auguuaaugaucugacuuugaag...	21	0	LT-2
...auguuaaugaucugacuuugaag...	9	0	JCK-3
...auguuaaugaucugacuuugaag...	23	0	LT-3
...auguuaaugaucugacuuugaagg...	178	0	JCK-1
...auguuaaugaucugacuuugaNgg...	2	1	LT-2
...auguuaaugaucugacuuuNaagg...	1	1	JCK-1
...auguuCaugaucugacuuugaagg...	3	1	LT-2
...aCguuaaugaucugacuuugaagg...	1	1	LT-2
...auguuaCugaucugacuuugaagg...	4	1	LT-2
...Cuguuaaugaucugacuuugaagg...	4	1	LCK-3
...auguuaaugaucugacuuugCagg...	2	1	JT-1
...auguuaaugCucugacuuugaagg...	1	1	LT-1
...auguuCaugaucugacuuugaagg...	1	1	JCK-1
...auguuaaugaucugCcuuugaagg...	1	1	LCK-2
...auguuaaugaucugacuuugaagg...	1112	0	LT-2
...auAuuaaugaucugacuuugaagg...	1	1	LCK-2
...auguuaaugCucugacuuugaagg...	1	1	LCK-2
...auNuuaaugaucugacuuugaagg...	4	1	LT-2
...auguuaCugaucugacuuugaagg...	1	1	LCK-3
...Cuguuaaugaucugacuuugaagg...	2	1	JCK-1
...Guguuaaugaucugacuuugaagg...	1	1	LCK-3
...auguuaaugaucugCcuuugaagg...	1	1	LT-1
...Cuguuaaugaucugacuuugaagg...	21	1	LCK-2
...aNguuaaugaucugacuuugaagg...	1	1	LCK-3
...auguuCaugaucugacuuugaagg...	2	1	LT-1
...auguuaaugaucuNacuuugaagg...	3	1	LCK-1
...Cuguuaaugaucugacuuugaagg...	5	1	LT-2
...auguuaaugCucugacuuugaagg...	1	1	LT-2
...auguuaCugaucugacuuugaagg...	2	1	LT-3
...auguuaaugaucugacuuugaagg...	587	0	LCK-1
...auguuaaugaucugacuuugaagg...	57	0	JT-2
...aNguuaaugaucugacuuugaagg...	1	1	JCK-2
...auguuaaugaucugacuuugaagg...	43	0	JT-3
...auguuCaugaucugacuuugaagg...	1	1	LCK-2
...auguuaaugaucugacuuugaagg...	114	0	JT-1
...auguuaaugaucugacuuugaagg...	531	0	LT-1
...auguuaaugaucugacuuugaCgg...	2	1	LT-2
...auguuaaugaucugCcuuugaagg...	1	1	LT-2
...augCuaaugaucugacuuugaagg...	1	1	LT-2
...auNuuaaugaucugacuuugaagg...	1	1	LCK-1
...auguuaaugaucugacuuugaagg...	1503	0	LCK-3
...auguuaaugaucugacuuugaagg...	205	0	JCK-3
...auguuaaugaucugacuuugaagg...	610	0	LCK-2
...Cuguuaaugaucugacuuugaagg...	1	1	LT-1
...auguuaaugaucugacuuCgaagg...	1	1	LT-1
...Cuguuaaugaucugacuuugaagg...	33	1	LCK-1
...auguuNaugaucugacuuugaagg...	1	1	LCK-1
...auguuaaugaucugNcuuugaagg...	2	1	LCK-1
...auguuaaugaucugacuuugaagA...	3	1	LT-3
...auguuaaugaucugacCuugaagg...	4	1	LT-2
...auguuaaugaucugacuuugaagg...	234	0	JCK-2
...auguuaaugaucugCcuuugaagg...	1	1	JCK-3
...auguuaaugGucugacuuugaagg...	1	1	JCK-2
...auguuaCugaucugacuuugaagg...	1	1	JCK-1
...auguuaaugaucugacuuugaagg...	429	0	LT-3
...auguuaaugauNugacuuugaagg...	1	1	JCK-1
...auguuaaugaucugacuuugaaUg...	1	1	JCK-2
...Cuguuaaugaucugacuuugaagg...	1	1	LT-3
...auguuaCugaucugacuuugaagg...	2	1	LT-1

Star Mature

uucgcgcagccccuguccaauucuaguuucaauuuuuauugugucacaauuaauucgaacggaacaacguggcaacuguucaauucugauuucaauuuuuuuuguuacauaauuaguucaaauaaaacaauuugacuacuguuuucuguuguagauuucuuagucauguuaaucuuuuuggcuuauucuuccagugaucgaccauguuaaugaucugacuuugaaggcuauuuagggcacuuaaauauau

aCguuaaugaucugacuuugaagg	1	1	JCK-3
auguuNaugaucugacuuugaaggc	1	1	LCK-1
Cuguuaaugaucugacuuugaaggc	5	1	JCK-1
auguuaaugaucugacuuugaaggA	21	1	LCK-1
auguuCaugaucugacuuugaaggc	2	1	JCK-3
auguuaaugaucugacuuugaaggA	44	1	LT-2
auguuaaugaucugacuuugaaggA	23	1	LCK-2
auguuaaugCucugacuuugaaggc	1	1	LT-2
auguuaaugaucugacuuugaagAc	1	1	JT-1
auguuaaugaucugacuuugaCggc	1	1	LT-2
auguuaaugaucugacuuugaaggU	16	1	LCK-3
auguuaaugaucugacuuuUaaggc	1	1	LT-1
auguuaaugaucugacCuugaaggc	3	1	LT-2
auguuaaugCucugacuuugaaggc	1	1	LCK-2
auguuCaugaucugacuuugaaggc	1	1	LCK-1
auguCaaugaucugacuuugaaggc	1	1	JCK-1
aNguuaaugaucugacuuugaaggc	1	1	JCK-2
auguuCaugaucugacuuugaaggc	1	1	LT-3
auguuaaugaucugacuuugaaggG	1	1	LT-3
auguuaaugCucugacuuugaaggc	1	1	LCK-1
auguuaaugaucugacuuugaaggA	21	1	LT-3
auguuaaugaucugacuuugaaggc	745	0	LCK-3
auguuaaCgaucugacuuugaaggc	1	1	LT-2
auguuaaugaucugCcuuugaaggc	1	1	LT-2
auguuaaugaucugacuuugCaggc	1	1	JCK-2
auguuaaugaucugacuuugaaggc	589	0	JCK-2
Guguuaaugaucugacuuugaaggc	1	1	LT-1
auguuaCugaucugacuuugaaggc	3	1	JCK-2
auguCaaugaucugacuuugaaggc	2	1	JCK-3
Cuguuaaugaucugacuuugaaggc	1	1	JT-1
auguuCaugaucugacuuugaaggc	1	1	JCK-2
auguuaCugaucugacuuugaaggc	2	1	LT-2
auguuGaugaucugacuuugaaggc	1	1	JCK-1
auguuaaugaucugacuuugaaggc	475	0	LT-2
auguuaaCgaucugacuuugaaggc	1	1	LCK-1
Cuguuaaugaucugacuuugaaggc	1	1	LCK-3
auguuaaugaucugacuuugaaggc	39	0	JT-2
auUuuaaugaucugacuuugaaggc	1	1	LCK-2
auguuaaugaucugacuuugaaggA	24	1	JCK-3
auguuaaugaucugacuuugaaggc	363	0	JCK-3
auguuaCugaucugacuuugaaggc	1	1	JCK-3
auguuaaugaucugacuuugaaggA	17	1	LT-1
auguuaCugaucugacuuugaaggc	2	1	LCK-2
auguuaaugaucugacuuugaaggU	3	1	LT-2
auguuaaugauNugacuuugaaggc	1	1	JCK-1
auguuaaugaucugCcuuugaaggc	1	1	LCK-1
auguuaaugaucugacuuugaaggA	7	1	JT-1
auguuaaugCucugacuuugaaggc	1	1	LCK-3
auguCaaugaucugacuuugaaggc	1	1	JCK-2
auguuaCugaucugacuuugaaggc	1	1	LT-3
auguuaaugGucugacuuugaaggc	1	1	JT-1
Cuguuaaugaucugacuuugaaggc	1	1	LCK-1
auguuaaugaucugacuuugaaggc	220	0	LCK-2
auguuaaugaucugNcuuugaaggc	4	1	LCK-1
auAuuaaugaucugacuuugaaggc	1	1	LT-2
auguuaaugaucugacuuugaaggA	47	1	LCK-3
auguuaaugaucugacuCugaaggc	1	1	JCK-2
auguuaaugaucugacuuugaaggA	22	1	JCK-1
auguuaaugauAugacuuugaaggc	1	1	LT-2
auguuCaugaucugacuuugaaggc	1	1	LCK-2
auguuCaugaucugacuuugaaggc	2	1	LT-2
auguuaaugaucugacuuugaaggc	264	0	LT-3
auguuaaugaucugacuuugaaggc	54	0	JT-3
auguuaaugaucugacuuugaaggc	206	0	LT-1
auNuuaaugaucugacuuugaaggc	2	1	LT-2
auguuaaugaucugacuuugaagNc	1	1	LT-2
auguuaauAaucugacuuugaaggc	1	1	LT-3
auguuaaugaucugacuuugaaggc	415	0	JCK-1
Cuguuaaugaucugacuuugaaggc	4	1	JCK-2
auguuaaugaucugacuuugaaggc	163	0	JT-1

Star Mature

uucgcgcagccccuguccaauucuaguuucaauuuuuauuguguccacaauuaauucgaacggaacaacguggcaacuguucaauucugauuucaauuuuuuuuguuacauaauuaguucaaauaaaacaauuugacuacuguuuucuguuguagauuucuuagucauguuaaucuuuuuggcuuauucuuccagugaucgaccauguuaaugaucugacuuugaaggcuauuuagggcacuuaaauauau

auguuaaugaucugacuuugaaggc	222	0	LCK-1
auguuaaugaucugacuuugaaggA	17	1	JCK-2
auguuaaugaucugacuuugaagUc	1	1	LCK-2
auguuCaugaucugacuuugaaggc	1	1	JCK-1
auguuaaugaucugacuuugaaggcu	117	0	LCK-2
auguuaaugaucugacuuugaaggcu	50	0	LT-3
auguuaaugaucugacuuugaaggcu	3	0	JT-3
auguuaaugaucugacuuugaaggcC	2	1	LT-3
auguuaaugaucugacuuCgaaggcu	1	1	LT-2
auguuaaugaucugacuuugaaggcA	2	1	LT-2
auguuaaugaucugacuuugaaggcu	71	0	JCK-1
Cuguuaaugaucugacuuugaaggcu	2	1	LT-2
auguuaCugaucugacuuugaaggcu	1	1	LT-3
auguuaaugaucugacuuugaaggcA	24	1	LCK-1
auguuaCugaucugacuuugaaggcu	1	1	LCK-3
auguuaaugaucugacuuugaaggcu	9	0	JT-1
auguuaaugaucugacuuugaaggcC	2	1	JCK-2
auguuCaugaucugacuuugaaggcu	2	1	LT-2
auguuaaugaucugacuuugaaggcA	20	1	LCK-2
auguuaaugaucugacuuugaaggcA	3	1	LT-3
auguuaaugaucugacuuugaaggcu	76	0	LT-1
aNguuaaugaucugacuuugaaggcu	2	1	JCK-2
auguuaaugaucugacuuugaaggcA	1	1	JCK-2
auguuaaugaucugacuuugaaggcA	1	1	LT-1
auguuaaugaucugacuuugaaggcu	90	0	JCK-3
auguuaaCgaucugacuuugaaggcu	1	1	LT-1
auguuaaugaucugacuuugaaggcG	1	1	LT-1
auguuaaugaucugacuuugaaggcu	108	0	JCK-2
auguuaCugaucugacuuugaaggcu	2	1	LT-2
auguuaaugaucugacuuugaaggcu	96	0	LCK-1
auguuaaugaucugacuuugaaggcu	172	0	LCK-3
auguuaaugaucugacuuugaaggcC	4	1	LT-1
auguuCaugaucugacuuugaaggcu	1	1	LCK-2
auAuuaaugaucugacuuugaaggcu	1	1	LCK-1
auguuaCugaucugacuuugaaggcu	1	1	JCK-1
auguuaaugaucugacuuugaaggcC	2	1	LT-2
auguCaaugaucugacuuugaaggcu	1	1	JT-1
aNguuaaugaucugacuuugaaggcu	1	1	JCK-1
auguuaaugaucugacuuugaaggcu	146	0	LT-2
auguuaaugaucugacuuugaaggcC	1	1	JCK-3
auguuaaugaucugacuuugaaggcA	2	1	JCK-3
auguuaaugaucugacuuugaaggcua	3	0	JT-2
auguuaaugaucugacuuugaaggcua	18	0	JCK-1
auguuaaugaucugacuuugaaggcua	6	0	JT-1
Cuguuaaugaucugacuuugaaggcua	1	1	LT-2
auguuaaugaucugacuuugaaggcua	51	0	JCK-2
auguuaCugaucugacuuugaaggcua	1	1	LT-3
auguuaaugaucugacuuugaCggcua	1	1	LT-3
auguuaaugauNugacuuugaaggcua	1	1	JT-1
auguuaCugaucugacuuugaaggcua	1	1	JCK-1
auguuaaugaucugCcuuugaaggcua	2	1	JCK-2
auguuaaugaucugacuuugaaggcua	72	0	LT-2
aNguuaaugaucugacuuugaaggcua	1	1	JCK-3
auguuaaugaucugacuuugaaggcuG	1	1	LT-2
auguuaaugaucuAacuuugaaggcua	1	1	LT-2
auguuaaugaucugacuuugaaggcua	80	0	LT-1
auguuaaugaucugacuuugaaggcua	131	0	LCK-3
auguuaauAaucugacuuugaaggcua	1	1	LCK-3
auguuaaugaucugacuuugaaggcua	58	0	LT-3
auguuCaugaucugacuuugaaggcua	1	1	LT-1
auguuaaugaucugacuuugaaggcua	23	0	JCK-3
auguuaaugaucugacuuugaCggcua	1	1	LT-2
auguuaaugaucugacuuugaaggcua	1	0	JT-3
auguuaaugaucugacuuugaaggcuCu	1	1	LCK-3
auguuaaugaucugacuuugaaggcuau	5	0	LT-3
auguuaaugaucugacuuugaaggcuau	18	0	LCK-1
auguuaaugaucugacuuugaaggcuau	20	0	LT-2
auguuaaugaucugacuuugaaggcuau	5	0	LT-1
auguuaaugaucugacuuugaaggcuaA	6	1	LCK-3
auguuaaugaucugacuuugaaggcuau	16	0	LCK-2

Star Mature

```
uucgcgcagccccuguccaauucuaguuucaauuuuuauugugucacaauuaauucgaacggaacaacguggcaacuguucaauucugauuucaauuuuuuuuguuacauaauuaguucaaauaaaacaauuugacuacuguuuucuguuguagauuucuuagucauguuaaucuuuuuggcuuauucuuccagugaucgaccauguuaaugaucugacuuugaaggcuauuuagggcacuuaaauauau
...........................................................................................................................................................................................................auguuaaugaucugacuuugaaggcuau...................  10    0  JCK-3
...........................................................................................................................................................................................................auguuaaugaucugacuuugaaggcuau...................  49    0  LCK-3
...........................................................................................................................................................................................................auguuaaugaucugacuuugaaggcuau...................  1     0  JCK-1
...........................................................................................................................................................................................................auguuaaugaucugacuuugaaggcuau...................  5     0  JCK-2
...........................................................................................................................................................................................................auguuaaugaucugacuCugaaggcuau...................  1     1  LCK-1
...........................................................................................................................................................................................................auguuaaugaucugacuuugaaggcuauG..................  3     1  LCK-3
...........................................................................................................................................................................................................auguuaaugaucugacuuugaaggcuauu..................  1     0  LT-3
...........................................................................................................................................................................................................auguuaaugaucugacuuugaaggcuauA..................  14    1  LCK-3
...........................................................................................................................................................................................................auguuaaugaucugacuuugaaggcuauA..................  2     1  JCK-1
...........................................................................................................................................................................................................auguuaaugaucugacuuugaaggcuauu..................  2     0  JCK-1
...........................................................................................................................................................................................................auguuaaugaucugacuuugaaggcuauA..................  2     1  LT-1
...........................................................................................................................................................................................................auguuaaugaucugacuuugaaggcuauu..................  2     0  LT-2
...........................................................................................................................................................................................................auguuaaugaucugacuuugaaggcuauA..................  2     1  LT-3
...........................................................................................................................................................................................................auguuaaugaucugacuuugaaggcuauu..................  8     0  JCK-2
...........................................................................................................................................................................................................auguuaaugaucugacuuugaaggcuauA..................  1     1  JCK-3
...........................................................................................................................................................................................................auguuaaugaucugacuuugaaggcuauu..................  3     0  LT-1
...........................................................................................................................................................................................................auguuaaugaucugacuuugaaggcuauu..................  12    0  LCK-3
...........................................................................................................................................................................................................auguuaaugaucugacuuugaaggcuauuA.................  2     1  JT-3
...........................................................................................................................................................................................................auguuaaugaucugacuuugaaggcuauuG.................  2     1  LCK-3
...........................................................................................................................................................................................................auguuaaugaucugacuuugaaggcuauuu.................  1     0  LT-3
...........................................................................................................................................................................................................auguuaaugaucugacuuugaaggcuauuA.................  1     1  LCK-3
............................................................................................................................................................................................................uguuaaugaucugacuuuga..........................  4     0  JCK-3
............................................................................................................................................................................................................uguuaaugaucugacuuuga..........................  1     0  LT-2
............................................................................................................................................................................................................uguuaaugaucugacuuuga..........................  3     0  JCK-1
............................................................................................................................................................................................................uguuaaugaucugacuuuga..........................  4     0  LCK-3
............................................................................................................................................................................................................uguuaaugaucugacuuuga..........................  4     0  LT-1
............................................................................................................................................................................................................uguuaaugaucugacuuugaa.........................  1     0  LCK-3
............................................................................................................................................................................................................uguuaaugaucugacuuugaa.........................  11    0  JCK-3
............................................................................................................................................................................................................uguuaaugaucugacuuugaa.........................  7     0  JCK-1
............................................................................................................................................................................................................uguuaaugaucugacuuugaa.........................  1     0  LT-3
............................................................................................................................................................................................................uguuaaugaucugacuuugaa.........................  14    0  JCK-2
............................................................................................................................................................................................................uguuaaugaucugacuuugaag........................  10    0  JCK-1
............................................................................................................................................................................................................uguuaaugaucugacuuugaag........................  10    0  JCK-2
............................................................................................................................................................................................................uguuaaugaucugacuuugaag........................  22    0  LT-1
............................................................................................................................................................................................................uguuaaugaucugacuuugaag........................  10    0  JCK-3
............................................................................................................................................................................................................uguuCaugaucugacuuugaag........................  1     1  LT-2
............................................................................................................................................................................................................ugNuaaugaucugacuuugaag........................  1     1  LCK-3
............................................................................................................................................................................................................uguuaaugaucugacuuugaag........................  54    0  LT-2
............................................................................................................................................................................................................uguuaaugaucugacuuugaag........................  24    0  LT-3
............................................................................................................................................................................................................uguuaaugaucugacuuugaGg........................  1     1  LT-2
............................................................................................................................................................................................................uguuaaugaucugacuuugaag........................  11    0  JT-1
............................................................................................................................................................................................................uguuaaugaucugacuuugaag........................  40    0  LCK-3
............................................................................................................................................................................................................uguuaCugaucugacuuugaag........................  1     1  LT-2
............................................................................................................................................................................................................uguuaaugaucugacuuugaCgg.......................  1     1  LT-2
............................................................................................................................................................................................................uguuCaugaucugacuuugaagg.......................  3     1  LT-1
............................................................................................................................................................................................................uNuuaaugaucugacuuugaagg.......................  1     1  JCK-3
............................................................................................................................................................................................................ugNuaaugaucugacuuugaagg.......................  6     1  LT-2
............................................................................................................................................................................................................uguuaCugaucugacuuugaagg.......................  2     1  LCK-2
............................................................................................................................................................................................................uguuaaugaucugacuuugaagg.......................  1014  0  LT-1
............................................................................................................................................................................................................uguuaaugCucugacuuugaagg.......................  1     1  LT-3
............................................................................................................................................................................................................ugCuaaugaucugacuuugaagg.......................  2     1  LT-2
............................................................................................................................................................................................................uguuaaugaucugacuuugaagg.......................  533   0  JCK-1
............................................................................................................................................................................................................uguuaaugauAugacuuugaagg.......................  1     1  LT-3
............................................................................................................................................................................................................uguuaaugaucugCcuuugaagg.......................  1     1  LCK-3
............................................................................................................................................................................................................uguuaCugaucugacuuugaagg.......................  2     1  LCK-1
............................................................................................................................................................................................................uguuCaugaucugacuuugaagg.......................  2     1  JT-3
............................................................................................................................................................................................................uguuaaugaucugacuuuAaagg.......................  1     1  LT-2
............................................................................................................................................................................................................uguuaaugaucugacuuugaGgg.......................  1     1  JCK-1
............................................................................................................................................................................................................uguuaCugaucugacuuugaagg.......................  2     1  LCK-3
............................................................................................................................................................................................................uguuaaugaucugacuuugaagg.......................  221   0  JT-3
............................................................................................................................................................................................................uguuaaugaucugCcuuugaagg.......................  1     1  JT-3
............................................................................................................................................................................................................ugGuaaugaucugacuuugaagg.......................  1     1  LT-2
............................................................................................................................................................................................................uguuaaugaucugacCuugaagg.......................  1     1  JT-1
............................................................................................................................................................................................................uguuaaugaucugacuuugaaAg.......................  1     1  LCK-3
............................................................................................................................................................................................................uguuaaugaucugacuuugaaAg.......................  1     1  LT-3
............................................................................................................................................................................................................uguuaCugaucugacuuugaagg.......................  9     1  LT-2
............................................................................................................................................................................................................uguuaaugaucugacuuugCagg.......................  3     1  LCK-2
............................................................................................................................................................................................................uguuaaugaucugCcuuugaagg.......................  2     1  JT-1
............................................................................................................................................................................................................uguuaaugaucugacuuugCagg.......................  2     1  LT-2
............................................................................................................................................................................................................uguuCaugaucugacuuugaagg.......................  6     1  LCK-2
```

Star Mature

```
uucgcgcagccccuguccaauucuaguuucaauuuuuauugugucacaauuaauucgaacggaacaacguggcaacuguucaauucugauuucaauuuuuuuuguuacauaauuaguucaaauaaaacaauuugacuacuguuuucuguuguagauuucuuagucauguuaaucuuuuuggcuuauucuuccagugaucgaccauguuaaugaucugacuuugaaggcuauuuagggcacuuaaauauau
............................................................................................................................................................................................................uguuaaugGucugacuuugaagg.......................   1      1      LCK-2
............................................................................................................................................................................................................uguuaaugaucugacCuugaagg.......................   1      1      LT-2
............................................................................................................................................................................................................uguuaauAaucugacuuugaagg.......................   2      1      LT-1
............................................................................................................................................................................................................uguuaaugaucugacuuugaagg.......................   3634   0      LCK-3
............................................................................................................................................................................................................uAuuaaugaucugacuuugaagg.......................   1      1      JCK-3
............................................................................................................................................................................................................uguuaaugaucugacuuugaagg.......................   1151   0      LCK-2
............................................................................................................................................................................................................uguuaaugaucugCcuuugaagg.......................   1      1      JCK-1
............................................................................................................................................................................................................uguuaaugaucugaAuuugaagg.......................   1      1      LCK-2
............................................................................................................................................................................................................uguuCaugaucugacuuugaagg.......................   3      1      JT-1
............................................................................................................................................................................................................uguuaaugaucugacuGugaagg.......................   1      1      LCK-1
............................................................................................................................................................................................................uguuCaugaucugacuuugaagg.......................   10     1      LCK-3
............................................................................................................................................................................................................uNuuaaugaucugacuuugaagg.......................   1      1      LCK-1
............................................................................................................................................................................................................uguuaaugaucugacuuugaagU.......................   2      1      LT-3
............................................................................................................................................................................................................uguuaaugaucugacuuugaagg.......................   1218   0      LCK-1
............................................................................................................................................................................................................uguuCaugaucugacuuugaagg.......................   3      1      JCK-3
............................................................................................................................................................................................................uAuuaaugaucugacuuugaagg.......................   2      1      LCK-2
............................................................................................................................................................................................................uguuaaugaucugacuuugaCgg.......................   2      1      JCK-1
............................................................................................................................................................................................................uguuaaugaucugacuuugCagg.......................   1      1      LT-3
............................................................................................................................................................................................................uguuaaugaucugacuCugaagg.......................   1      1      JT-1
............................................................................................................................................................................................................uguuaaugaucugacuuugaagA.......................   4      1      LT-2
............................................................................................................................................................................................................uguuaaugaucugacuuugaagg.......................   166    0      JT-2
............................................................................................................................................................................................................uguuaaugCucugacuuugaagg.......................   1      1      LCK-3
............................................................................................................................................................................................................uguuaaugaucugNcuuugaagg.......................   4      1      LCK-1
............................................................................................................................................................................................................uguuaaugCucugacuuugaagg.......................   1      1      JT-1
............................................................................................................................................................................................................Cguuaaugaucugacuuugaagg.......................   1      1      LT-1
............................................................................................................................................................................................................uguuaaugaucugaNuuugaagg.......................   1      1      LCK-1
............................................................................................................................................................................................................uguuaaugaucugacuuugaagg.......................   1065   0      LT-3
............................................................................................................................................................................................................uguuaCugaucugacuuugaagg.......................   4      1      LT-1
............................................................................................................................................................................................................uguuaaugaucugacuuugaCgg.......................   1      1      JT-3
............................................................................................................................................................................................................uguuaaugauUugacuuugaagg.......................   1      1      LT-3
............................................................................................................................................................................................................uguuaaugGucugacuuugaagg.......................   1      1      LT-1
............................................................................................................................................................................................................uAuuaaugaucugacuuugaagg.......................   6      1      LT-2
............................................................................................................................................................................................................uAuuaaugaucugacuuugaagg.......................   1      1      LT-3
............................................................................................................................................................................................................Gguuaaugaucugacuuugaagg.......................   1      1      JCK-2
............................................................................................................................................................................................................uguuaaugaucugacuuugCagg.......................   1      1      JCK-3
............................................................................................................................................................................................................uNuuaaugaucugacuuugaagg.......................   1      1      JCK-2
............................................................................................................................................................................................................uguuaaugaCcugacuuugaagg.......................   1      1      LCK-3
............................................................................................................................................................................................................uguuaauAaucugacuuugaagg.......................   2      1      LT-3
............................................................................................................................................................................................................uAuuaaugaucugacuuugaagg.......................   1      1      JCK-1
............................................................................................................................................................................................................uguuaaugaucugCcuuugaagg.......................   3      1      LT-2
............................................................................................................................................................................................................uguuaaugCucugacuuugaagg.......................   1      1      LCK-1
............................................................................................................................................................................................................uguCaaugaucugacuuugaagg.......................   1      1      JCK-1
............................................................................................................................................................................................................uguuCaugaucugacuuugaagg.......................   1      1      LT-3
............................................................................................................................................................................................................uguuaGugaucugacuuugaagg.......................   1      1      LCK-3
............................................................................................................................................................................................................uguuaaugaucugaUuuugaagg.......................   1      1      LT-2
............................................................................................................................................................................................................uguuaaugaucugacCuugaagg.......................   1      1      LCK-2
............................................................................................................................................................................................................uguuaaugaucugacuuugaCgg.......................   2      1      LCK-3
............................................................................................................................................................................................................uguuaaugaucugaAuuugaagg.......................   1      1      LCK-3
............................................................................................................................................................................................................uguuaaugaucuAacuuugaagg.......................   1      1      LT-2
............................................................................................................................................................................................................uguuaaugaucugGcuuugaagg.......................   1      1      JT-3
............................................................................................................................................................................................................uguuaaugaucugaAuuugaagg.......................   1      1      JCK-3
............................................................................................................................................................................................................uguuaaugaucugacuuugaagg.......................   2271   0      LT-2
............................................................................................................................................................................................................uguuaNugaucugacuuugaagg.......................   3      1      LCK-1
............................................................................................................................................................................................................uAuuaaugaucugacuuugaagg.......................   2      1      LT-1
............................................................................................................................................................................................................uguNaaugaucugacuuugaagg.......................   1      1      JCK-1
............................................................................................................................................................................................................Cguuaaugaucugacuuugaagg.......................   1      1      JT-1
............................................................................................................................................................................................................uguuaauAaucugacuuugaagg.......................   1      1      JCK-3
............................................................................................................................................................................................................uguuaaugaucugacCuugaagg.......................   5      1      LT-3
............................................................................................................................................................................................................uguuaaugaucugacuuugCagg.......................   1      1      LCK-1
............................................................................................................................................................................................................uguuaaugaucugCcuuugaagg.......................   1      1      LCK-1
............................................................................................................................................................................................................uguuaaugCucugacuuugaagg.......................   8      1      LT-2
............................................................................................................................................................................................................uguuaaugaucugacuuugaGgg.......................   2      1      LCK-1
............................................................................................................................................................................................................uguuaaugauUugacuuugaagg.......................   2      1      LT-2
............................................................................................................................................................................................................uAuuaaugaucugacuuugaagg.......................   1      1      JCK-2
............................................................................................................................................................................................................uguuaaugaucugacuuugaagg.......................   437    0      JCK-3
............................................................................................................................................................................................................uguuaauAaucugacuuugaagg.......................   2      1      LT-2
............................................................................................................................................................................................................uguuaaugauUugacuuugaagg.......................   2      1      JCK-1
............................................................................................................................................................................................................uAuuaaugaucugacuuugaagg.......................   5      1      LCK-3
............................................................................................................................................................................................................uguuaaugaucugacuuugaagg.......................   810    0      JT-1
............................................................................................................................................................................................................uguuCaugaucugacuuugaagg.......................   1      1      JCK-1
```

Star Mature

uucgcgcagccccuguccaauucuaguuucaauuuuuauugugucacaauuaauucgaacggaacaacguggcaacuguucaauucugauuucaauuuuuuuuguuacauaauuaguucaaauaaaacaauuugacuacuguuuucuguuguagauuucuuagucauguuaaucuuuuuggcuuauucuuccagugaucgaccauguuaaugaucugacuuugaaggcuauuuagggcacuuaaauauau

uguuCaugaucugacuuugaagg	3	1	LCK-1
uguuaaugaucugacuuugaagg	706	0	JCK-2
uguuCaugaucugacuuugaagg	20	1	LT-2
uguuaaugCucugacuuugaaggc	5	1	LT-2
uguuCaugaucugacuuugaaggc	3	1	JCK-3
uguuaaugaucugacuuugNaggc	1	1	JCK-1
uguuaaugaCcugacuuugaaggc	1	1	LT-2
uguuaaugaucugacuuugaaggU	1	1	JCK-3
uguuaaugaCcugacuuugaaggc	2	1	JCK-3
uguuaaugaucugaGuuugaaggc	2	1	LCK-1
uguuaaugaucugacuuugaaggc	1492	0	JCK-1
uguuCaugaucugacuuugaaggc	8	1	LCK-3
uguuaaCgaucugacuuugaaggc	1	1	LCK-3
uguuaaugaucugacuuugaaggc	988	0	LCK-2
uguuaaugaucugacuuugaaggU	11	1	LCK-3
uguuaaugaucugacuuugaaggc	999	0	LCK-1
uguuaauAaucugacuuugaaggc	1	1	LT-1
uguuaaugCucugacuuugaaggc	6	1	JCK-2
uguuaaugauUugacuuugaaggc	1	1	JCK-1
uguuaaugaucugacuuugaaggA	70	1	JCK-2
uguuaCugaucugacuuugaaggc	1	1	LT-1
uguuCaugaucugacuuugaaggc	5	1	JCK-2
uguuaNugaucugacuuugaaggc	1	1	LCK-1
ugCuaaugaucugacuuugaaggc	1	1	LT-2
uUuuaaugaucugacuuugaaggc	1	1	JCK-1
Cguuaaugaucugacuuugaaggc	1	1	JCK-3
uguuaaugaucugacuuugCaggc	1	1	LT-3
uguuaaugaucugacuuugaaggc	655	0	LT-1
uguuaaugaucugacuCugaaggc	1	1	LCK-3
uguuaaugaucugacuuuAaaggc	1	1	JCK-2
uguuaaugaucugacuuuUaaggc	1	1	JCK-2
uguuCaugaucugacuuugaaggc	1	1	JT-1
uguuaaugaucugacuuugaaggc	922	0	LT-3
uguuaaugaucugacuuugaaggA	61	1	LT-3
uguuaaugaucugacuuugaaggN	3	1	LT-2
uguuaaugaucCgacuuugaaggc	1	1	JCK-2
uguuaaugaucugCcuuugaaggc	1	1	JCK-1
uguuCaugaucugacuuugaaggc	2	1	JCK-1
uguuaaugCucugacuuugaaggc	1	1	LT-3
uUuuaaugaucugacuuugaaggc	2	1	LT-2
uguuaaugauUugacuuugaaggc	2	1	JCK-2
uguuaaugCucugacuuugaaggc	1	1	JT-1
uguuaaugaucugacuuugaaggA	124	1	LT-2
uguuaaugaucugaNuuugaaggc	3	1	LCK-1
uguuaaugaucugNcuuugaaggc	1	1	LCK-1
uguuaaugaucugacuuuAaaggc	1	1	LT-2
uguuaaugaucugCcuuugaaggc	2	1	JT-1
uguuaaugaucugacuuugaaggU	3	1	JCK-2
uguuaaugaucugacuuugaaggA	54	1	JCK-3
uAuuaaugaucugacuuugaaggc	6	1	LT-2
uguuaGugaucugacuuugaaggc	1	1	JCK-2
uguuaaugaucugGcuuugaaggc	1	1	JCK-2
ugCuaaugaucugacuuugaaggc	1	1	LCK-3
uguuCaugaucugacuuugaaggc	2	1	LT-1
uguuaaugaucugCcuuugaaggc	3	1	LT-2
uguuaaugCucugacuuugaaggc	3	1	LCK-3
uguuaCugaucugacuuugaaggc	7	1	LT-2
uguuaaugaucugacuuugaCggc	2	1	LCK-3
uguuaaugaucugaAuuugaaggc	1	1	LT-2
uCuuaaugaucugacuuugaaggc	1	1	LT-2
uguuaauUaucugacuuugaaggc	1	1	LT-2
uguuaaugaucugacuuugaaggc	584	0	JT-1
uUuuaaugaucugacuuugaaggc	1	1	LCK-3
uguuaaugaucugacuuugaaggA	17	1	JT-2
uguuaaugaucugacuuugaaggU	4	1	LT-3
uNuuaaugaucugacuuugaaggc	1	1	JCK-1
uguuaaugCucugacuuugaaggc	1	1	JCK-3
uguuaaugaucugacuuugCaggc	1	1	JCK-3
uguuaaugaucugacuuugaaggc	2036	0	JCK-2
uguuaaugaucugacuuuAaaggc	1	1	JT-1

Star Mature

uucgcgcagccccuguccaauucuaguuucaauuuuuauugugucacaauuaauucgaacggaacaacguggcaacuguucaauucugauuucaauuuuuuuuguuacauaauuaguucaaauaaaacaauuugacuacuguu uucuguuguagauuucuuag ucauguuaaucuuuuuggcuuauucuuccagugaucgacca uguuaaugaucugacuuugaagg cuauuuagggcacuuaaauauau			
uNuuaaugaucugacuuugaaggc	2	1	JT-1
uguuaaugaucugacuuugaaggA	6	1	JT-1
uguuaaugaucugacuuugaaggG	5	1	LCK-3
uNuuaaugaucugacuuugaaggc	3	1	JCK-3
uguuaaugaucugCcuuugaaggc	1	1	LCK-2
uNuuaaugaucugacuuugaaggc	2	1	JCK-2
uguuaaugaucugCcuuugaaggc	3	1	LCK-3
uguuCaugaucugacuuugaaggc	7	1	LT-2
uguuaCugaucugacuuugaaggc	3	1	LT-3
uguuaaugaCcugacuuugaaggc	1	1	LCK-1
uguuaaugaucugacuuugaCggc	1	1	JCK-2
uguuCaugaucugacuuugaaggc	1	1	JT-3
uguuaaugaucugacuuugaaNgc	2	1	LT-2
uguCaaugaucugacuuugaaggc	1	1	JT-1
uAuuaaugaucugacuuugaaggc	2	1	JCK-1
uguuaaugaucugacuuugaCggc	2	1	LT-2
uguuaaugaucugCcuuugaaggc	1	1	LT-3
uguuCaugaucugacuuugaaggc	5	1	LT-3
uguuaaugaucugGcuuugaaggc	1	1	LCK-1
uguuaCugaucugacuuugaaggc	4	1	LCK-3
uguuaaugaucugacuuugCaggc	1	1	LCK-1
uguuaCugaucugacuuugaaggc	4	1	JCK-2
uguuaaugaucugaGuuugaaggc	1	1	JT-2
ugCuaaugaucugacuuugaaggc	1	1	LT-3
uguuaaugCucugacuuugaaggc	1	1	LCK-1
uguuaaugaucugacuuugaCggc	1	1	LCK-2
uguuaaugaucugacCuugaaggc	1	1	JCK-1
uguuaaugaucugacuuugaaggA	41	1	JCK-1
uguuaaugaucugacuuugaaggc	2658	0	LCK-3
uguuaaugaCcugacuuugaaggc	1	1	JCK-2
uguuaaugaucugacuuugCaggc	2	1	LT-2
uguuaaugaCcugacuuugaaggc	1	1	JCK-1
ugNuaaugaucugacuuugaaggc	1	1	LCK-1
uguuaaugaucugacuuugaaggG	4	1	LT-2
uguuaaugCucugacuuugaaggc	3	1	JCK-1
uguuaaugaucugacuuugaaggc	1680	0	LT-2
uguuaaugauGugacuuugaaggc	1	1	JCK-1
uguuaaugGucugacuuugaaggc	1	1	LT-2
uguuaaugaucugacuuugaaggc	102	0	JT-2
uguuaaugCucugacuuugaaggc	3	1	LCK-2
uguuaauUaucugacuuugaaggc	1	1	JCK-1
uguuaaugaucugacuCugaaggc	1	1	LCK-1
uAuuaaugaucugacuuugaaggc	1	1	JCK-2
uAuuaaugaucugacuuugaaggc	2	1	LCK-3
uguuaaugaucNgacuuugaaggc	2	1	JCK-1
uguuaaugaucugacuuugaNggc	1	1	JCK-1
uguuaaugCucugacuuugaaggc	1	1	JT-3
uguuaaugaucugacuuugaagAc	1	1	LT-2
uguuaaugaucugacuuugaaCgc	1	1	JCK-2
uguuaaugaucugacuuugaaggA	76	1	LCK-2
uAuuaaugaucugacuuugaaggc	1	1	JT-1
uguuaaugaucugacuCugaaggc	1	1	LCK-2
uguuaCugaucugacuuugaaggc	2	1	JCK-3
uguuaaugaucugacuuugaaggG	1	1	LT-3
uguuaaugaucugCcuuugaaggc	1	1	LT-1
uguuaaugauUugacuuugaaggc	1	1	LT-1
uguuaaugaucugacuuugaaggc	1338	0	JCK-3
uguuCaugaucugacuuugaaggc	2	1	LCK-2
uguuaauAaucugacuuugaaggc	1	1	JT-1
uguuaaugaucugacuuugaaggA	20	1	JT-3
uguuaauAaucugacuuugaaggc	2	1	LT-2
uguuaaugaucugacuuugaaggc	161	0	JT-3
uguuaaugaucugacuuugaaggA	77	1	LCK-1
uAuuaaugaucugacuuugaaggc	1	1	LT-1
uguuaaugaucugacuuugaaggA	249	1	LCK-3
uguuaaugaucugacuuugCaggc	1	1	JCK-2
uguuaaugaucugacuuugaaggA	59	1	LT-1
uguuaaugaucugacuuugaaggU	2	1	LT-2
Cguuaaugaucugacuuugaaggc	1	1	LCK-1
uguuaaugaucugacCuugaaggc	3	1	JCK-2

```
                                                                                                                                                                                        Star                                                                Mature
uucgcgcagccccuguccaauucuaguuucaauuuuuauugugucacaauuaauucgaacggaacaacguggcaacuguucaauucugauuucaauuuuuuuuguuacauaauuaguucaaauaaaacaauuugacuacuguuuucuguuguagauuucuuagucauguuaaucuuuuuggcuuauucuuccagugaucgaccauguuaaugaucugacuuugaaggcuauuuagggcacuuaaauauau
............................................................................................................................................................................................................ugCuaaugaucugacuuugaaggc......................   1     1   JT-1
............................................................................................................................................................................................................uguuCaugaucugacuuugaaggc......................   1     1   LCK-1
............................................................................................................................................................................................................uguuaaugaucugacuuugaaggcC.....................   6     1   LCK-3
............................................................................................................................................................................................................uguuCaugaucugacuuugaaggcu.....................   1     1   LT-3
............................................................................................................................................................................................................uguuaaugaucugacuuugaaggcA.....................   9     1   LT-2
............................................................................................................................................................................................................uguuaaugaucugacuuugaaggcu.....................   668   0   LCK-3
............................................................................................................................................................................................................uguuaCugaucugacuuugaaggcu.....................   1     1   LT-1
............................................................................................................................................................................................................uguuaaugaucugacuuugaaggcu.....................   52    0   JT-1
............................................................................................................................................................................................................uguuaaugaucugacuuugaaggcG.....................   2     1   LCK-3
............................................................................................................................................................................................................uguuaaugaucugacuuugaCggcu.....................   1     1   LCK-3
............................................................................................................................................................................................................uAuuaaugaucugacuuugaaggcu.....................   1     1   LT-1
............................................................................................................................................................................................................uguuaaugaucugacuuugaaggcC.....................   1     1   JT-1
............................................................................................................................................................................................................uguuCaugaucugacuuugaaggcu.....................   1     1   LCK-3
............................................................................................................................................................................................................uguuCaugaucugacuuugaaggcu.....................   3     1   LT-2
............................................................................................................................................................................................................uguuaaugaucugacuuugaaggcu.....................   244   0   LT-1
............................................................................................................................................................................................................uguuaaugaucugacuuugaaggcA.....................   1     1   JCK-1
............................................................................................................................................................................................................uguuaaugaucugacuuugaaggUu.....................   4     1   JCK-2
............................................................................................................................................................................................................uguuaaugaucugacuuugaaggcG.....................   1     1   LT-2
............................................................................................................................................................................................................uguuaaugaucugacuuugaaggcC.....................   3     1   JCK-3
............................................................................................................................................................................................................uguuaaugaucugacuuugaaggcG.....................   4     1   JCK-3
............................................................................................................................................................................................................uguuaaugaucugacuuugCaggcu.....................   1     1   JCK-2
............................................................................................................................................................................................................uguuaaugaucugacuuugaaggcu.....................   206   0   LT-3
............................................................................................................................................................................................................uguuaaugaucugacuuugaaggcu.....................   51    0   LCK-1
............................................................................................................................................................................................................uguuaaugaucugacuuugNaggcu.....................   2     1   JCK-1
............................................................................................................................................................................................................uguuaaugaucugacuuugaaggcA.....................   1     1   JT-1
............................................................................................................................................................................................................uguuaCugaucugacuuugaaggcu.....................   1     1   JCK-3
............................................................................................................................................................................................................uguuaaugaucugacuuugaaggcu.....................   427   0   LT-2
............................................................................................................................................................................................................uguuaaugaucugacuuugaagNcu.....................   1     1   JCK-3
............................................................................................................................................................................................................uguuaaugCucugacuuugaaggcu.....................   2     1   JCK-2
............................................................................................................................................................................................................uguuaaugaucugacuuugaaggcG.....................   3     1   JCK-1
............................................................................................................................................................................................................Cguuaaugaucugacuuugaaggcu.....................   1     1   LT-3
............................................................................................................................................................................................................uguuaaugaucugacuCugaaggcu.....................   1     1   JT-1
............................................................................................................................................................................................................uguuCaugaucugacuuugaaggcu.....................   1     1   JCK-1
............................................................................................................................................................................................................uguuaaugaucugacuuugaaggcG.....................   4     1   JCK-2
............................................................................................................................................................................................................uguuaaugaucugacuuugaCggcu.....................   2     1   LT-2
............................................................................................................................................................................................................uguuaaugaucugacuuugaaggcC.....................   1     1   LCK-2
............................................................................................................................................................................................................uguuaaugaucugacuuugaaggcA.....................   2     1   JCK-2
............................................................................................................................................................................................................uguuaaugaucugacuuugaaggcA.....................   2     1   JT-2
............................................................................................................................................................................................................uguuaaugaucugacuuugaaggcC.....................   5     1   LT-3
............................................................................................................................................................................................................uguuaaugaucugacuuugaaggcA.....................   11    1   LCK-3
............................................................................................................................................................................................................uguuaaugCucugacuuugaaggcu.....................   2     1   LT-2
............................................................................................................................................................................................................uAuuaaugaucugacuuugaaggcu.....................   1     1   LT-2
............................................................................................................................................................................................................uguuaaugaucugacuuugaNggcu.....................   1     1   JCK-1
............................................................................................................................................................................................................uguuaaugaucugacuuugaaggcu.....................   5     0   JT-3
............................................................................................................................................................................................................uguuaCugaucugacuuugaaggcu.....................   1     1   LCK-3
............................................................................................................................................................................................................uguuaaugaucugacuuugaaggcu.....................   53    0   LCK-2
............................................................................................................................................................................................................ugNuaaugaucugacuuugaaggcu.....................   1     1   LT-2
............................................................................................................................................................................................................uguuaaugaucugacuuugaaggcu.....................   576   0   JCK-2
............................................................................................................................................................................................................uAuuaaugaucugacuuugaaggcu.....................   1     1   LT-3
............................................................................................................................................................................................................uguuaaugGucugacuuugaaggcu.....................   1     1   LCK-2
............................................................................................................................................................................................................uguuaaugaucugacuuugaaggcC.....................   3     1   JCK-2
............................................................................................................................................................................................................uguuaaugaucugacuuugaaggcA.....................   1     1   JT-3
............................................................................................................................................................................................................uguuaaugauUugacuuugaaggcu.....................   1     1   LT-3
............................................................................................................................................................................................................uguuaCugaucugacuuugaaggcu.....................   4     1   LT-2
............................................................................................................................................................................................................uguuaaugaucugCcuuugaaggcu.....................   1     1   LCK-3
............................................................................................................................................................................................................uguuaaugaucugacuuugaaggcA.....................   6     1   JCK-3
............................................................................................................................................................................................................uguuaaugaucugacuuugaaggcG.....................   5     1   LT-1
............................................................................................................................................................................................................uguuaaugaucugacuuugaaggcG.....................   2     1   LT-3
............................................................................................................................................................................................................uguuaaugaucugCcuuugaaggcu.....................   2     1   LT-2
............................................................................................................................................................................................................uguuaaugaucugacuuugaaggcC.....................   4     1   LT-1
............................................................................................................................................................................................................uguuaaugaucugacuuugaaggcC.....................   1     1   JCK-1
............................................................................................................................................................................................................uguuaaugaucugacuuugaaggcu.....................   390   0   JCK-3
............................................................................................................................................................................................................uguuaaugaucugacuuugaaggcu.....................   320   0   JCK-1
............................................................................................................................................................................................................uguuaaugaucugacuuugaaggcC.....................   3     1   LT-2
............................................................................................................................................................................................................uguuaaugaucugacuuugaaggcA.....................   5     1   LT-1
............................................................................................................................................................................................................uUuuaaugaucugacuuugaaggcu.....................   1     1   LT-2
............................................................................................................................................................................................................uguuaaugaucugacuuugaaggcu.....................   1     0   JT-2
............................................................................................................................................................................................................uguuaaugaucugacuuugaaggNu.....................   1     1   LT-2
............................................................................................................................................................................................................uguNaaugaucugacuuugaaggcu.....................   1     1   JCK-1
............................................................................................................................................................................................................uguuaaugaucugacuuugaaggcA.....................   4     1   LT-3
```

Star Mature

```
uucgcgcagccccuguccaauucuaguuucaauuuuuauugugucacaauuaauucgaacggaacaacguggcaacuguucaauucugauuucaauuuuuuuuguuacauaauuaguucaaauaaaacaauuugacuacuguuuucuguuguagauuucuuagucauguuaaucuuuuuggcuuauucuuccagugaucgaccauguuaaugaucugacuuugaaggcuauuuagggcacuuaaauauau
............................................................................................................................................................................................................uguuaCugaucugacuuugaaggcua......................	1	1	LCK-3
............................................................................................................................................................................................................uguuaaugaucugacuuCgaaggcua......................	1	1	LCK-3
............................................................................................................................................................................................................uguuaaugaucugacuuugCaggcua......................	2	1	LCK-1
............................................................................................................................................................................................................uguuaaugaucugacuuugaCggcua......................	1	1	LT-2
............................................................................................................................................................................................................uguuaCugaucugacuuugaaggcua......................	3	1	LT-2
............................................................................................................................................................................................................uguuaaugaucugacuuugaaNgcua......................	1	1	LT-2
............................................................................................................................................................................................................uguuaaugaucugacuuugaaggcua......................	3	0	JT-1
............................................................................................................................................................................................................uguuaaugaucugacuuugaaggcua......................	132	0	LT-1
............................................................................................................................................................................................................uguuaaugaucugCcuuugaaggcua......................	1	1	LT-3
............................................................................................................................................................................................................uAuuaaugaucugacuuugaaggcua......................	1	1	LT-3
............................................................................................................................................................................................................uguuaaugaucugacuuugaaggcuU......................	1	1	LT-1
............................................................................................................................................................................................................uguuCaugaucugacuuugaaggcua......................	2	1	LT-1
............................................................................................................................................................................................................uguuaaugaucugacuuugaaggcuU......................	2	1	LT-2
............................................................................................................................................................................................................uguuaaugaucugacuuugaaggAua......................	1	1	LCK-3
............................................................................................................................................................................................................uguuaCugaucugacuuugaaggcua......................	1	1	JCK-3
............................................................................................................................................................................................................uguuaaugaucugacuuugaaggcua......................	61	0	LCK-2
............................................................................................................................................................................................................ugNuaaugaucugacuuugaaggcua......................	1	1	LT-2
............................................................................................................................................................................................................uguuaaugaucugacCuugaaggcua......................	3	1	LT-2
............................................................................................................................................................................................................uguuaaugaucugacuuugaaggcua......................	1	0	JT-2
............................................................................................................................................................................................................uguuaaugaucugacuuugaaggcua......................	72	0	JCK-1
............................................................................................................................................................................................................uguuaaugaucugacuuugaaggcua......................	177	0	LT-2
............................................................................................................................................................................................................uguuaCugaucugacuuugaaggcua......................	1	1	JCK-2
............................................................................................................................................................................................................uguuaaugaucugacuuugaaggcua......................	74	0	JCK-3
............................................................................................................................................................................................................uguuaaugaucugacuuugaagAcua......................	1	1	LT-2
............................................................................................................................................................................................................uguuaaugaucugacuuugaaggcua......................	55	0	LCK-1
............................................................................................................................................................................................................uguuaaugaucugacuuugaaggcua......................	8	0	JT-3
............................................................................................................................................................................................................uguuaaugaucugacuuugaaggcua......................	111	0	LT-3
............................................................................................................................................................................................................uguuaGugaucugacuuugaaggcua......................	1	1	JCK-3
............................................................................................................................................................................................................uguuaaugaucugacuuugaaggcua......................	291	0	LCK-3
............................................................................................................................................................................................................uguuCaugaucugacuuugaaggcua......................	1	1	LT-2
............................................................................................................................................................................................................uguuCaugaucugacuuugaaggcua......................	2	1	LCK-3
............................................................................................................................................................................................................uguuaaugaucugacuuugaaggcua......................	77	0	JCK-2
............................................................................................................................................................................................................uguuaGugaucugacuuugaaggcua......................	1	1	JCK-1
............................................................................................................................................................................................................uguuaaugaucugCcuuugaaggcua......................	1	1	JCK-2
............................................................................................................................................................................................................uguuaaugaucugacuuugaaggcuau.....................	10	0	JCK-1
............................................................................................................................................................................................................uguuaaugauAugacuuugaaggcuau.....................	1	1	LCK-3
............................................................................................................................................................................................................uguuaaugaucugCcuuugaaggcuau.....................	1	1	LCK-3
............................................................................................................................................................................................................uguuCaugaucugacuuugaaggcuau.....................	1	1	LT-2
............................................................................................................................................................................................................uguuaaugaucugacuuugaaggcuau.....................	29	0	LT-2
............................................................................................................................................................................................................uguuaaugaucugacuuugaaggcuau.....................	8	0	JCK-3
............................................................................................................................................................................................................uguuCaugaucugacuuugaaggcuau.....................	1	1	LCK-1
............................................................................................................................................................................................................uguuaaugaucugacuuugaaggcuau.....................	18	0	JCK-2
............................................................................................................................................................................................................uguuaaugaucugacuuugaaggcuau.....................	19	0	LCK-1
............................................................................................................................................................................................................uguuaaugaucugacuuugaaggcuaA.....................	5	1	JCK-2
............................................................................................................................................................................................................uguuaaugaucugacuuugaaggcuaA.....................	2	1	LCK-3
............................................................................................................................................................................................................uguuaaugaucugacuuugaaggcuaA.....................	1	1	LT-2
............................................................................................................................................................................................................uguuaaugaucugacuuugaaggcuaA.....................	2	1	JCK-1
............................................................................................................................................................................................................uguuCaugaucugacuuugaaggcuau.....................	2	1	LCK-2
............................................................................................................................................................................................................uguuaaugaucugacuuugaaggcuau.....................	16	0	LCK-2
............................................................................................................................................................................................................uguuaaugaucugacuuugaaggcuau.....................	27	0	LT-1
............................................................................................................................................................................................................uguuaaugaucugacuuugaaggcuaA.....................	1	1	JCK-3
............................................................................................................................................................................................................uguuaaugaucugacuuugaaggcuau.....................	66	0	LCK-3
............................................................................................................................................................................................................uguuaaugaucugacuuugaaggcuau.....................	10	0	LT-3
............................................................................................................................................................................................................uguuaaugaucuAacuuugaaggcuau.....................	1	1	LT-2
............................................................................................................................................................................................................uguuaaugaucugacuuugaaggcuauA....................	2	1	JCK-1
............................................................................................................................................................................................................uguuaaugaucugacuuugaaggcuauG....................	4	1	LT-2
............................................................................................................................................................................................................uguuaaugaucugacuuugaaggcuauA....................	26	1	LCK-3
............................................................................................................................................................................................................uguuaaugaucugacCuugaaggcuauu....................	1	1	LT-3
............................................................................................................................................................................................................uguuaaugaucugacuuugaaggcuauA....................	9	1	LT-1
............................................................................................................................................................................................................uguuaaugaucugacuuugaaggcuauu....................	1	0	JT-3
............................................................................................................................................................................................................uguuaaugaucugacuuugaaggcuauu....................	8	0	JCK-3
............................................................................................................................................................................................................uguuaaugaucugacuuugaaggcuauA....................	3	1	JCK-3
............................................................................................................................................................................................................uguuaaugaucugacuuugaaggcuauG....................	1	1	LCK-3
............................................................................................................................................................................................................uguuaaugaucugacuuugaaggcuauu....................	27	0	LCK-3
............................................................................................................................................................................................................uguuaaugaucugacuuugaaggcuauu....................	14	0	LT-1
............................................................................................................................................................................................................uguuaaugaucugacuuugaaggcuauA....................	6	1	LT-2
............................................................................................................................................................................................................uguuCaugaucugacuuugaaggcuauu....................	1	1	LCK-3
............................................................................................................................................................................................................uguuaaugaucugacuuugaaggcuauu....................	14	0	JCK-2
............................................................................................................................................................................................................uguuaaugaucugacuuugaaggcuauu....................	16	0	JCK-1
............................................................................................................................................................................................................uguuaaugaucugacuuugaaggcuauu....................	8	0	LT-3
```

Star **Mature**

uucgcgcagccccuguccaauucuaguuucaauuuuuauugugucacaauuaauucgaacggaacaacguggcaacuguucaauucugauuucaauuuuuuuuguuacauaauuaguucaaauaaaacaauuugacuacuguuuucuguuguagauuucuuagucauguuaaucuuuuuggcuuauucuuccagugaucgaccauguuaaugaucugacuuugaaggcuauuuagggcacuuaaauauau			
uguuaaugaucugacuuugaaggcuauA	2	1	JCK-2
uguuaaugaucugacuuugaaggcuauu	9	0	LT-2
uguuaaugaucugacuuugaaggcuauA	3	1	LT-3
uguuaaugaucugacuuugaaggcuauuu	2	0	LCK-1
uguuaaugaucugacuuugaaggcuauuA	4	1	JCK-2
uguuaaugaucugacuuugaaggcuauuA	1	1	JCK-1
uguuaaugaucugacuuugaaggcuauuA	1	1	JCK-3
uguuaaugaucugacuuugaaggcuauuA	8	1	LCK-3
uguuaaugaucugacuuugaaggcuauuu	3	0	LCK-2
uguuaaugaucugacuuugaaggcuauuA	2	1	LT-2
uguuaaugaucugacuuugaaggcuauuu	1	0	JCK-2
guuaaugaucugacuuuga	1	0	LT-2
guuaaugaucugacuuugaag	1	0	LT-3
guuaaugaucugacuuugaag	1	0	JCK-1
guuaaugaucugacuuugaag	1	0	JCK-2
guuaaugaucugacuuugaag	1	0	JCK-3
guuCaugaucugacuuugaagg	2	1	LT-2
guuaaugaucugacuuugaagg	51	0	LT-3
guuaaugaucugacuuugaagg	133	0	LT-2
guuaaugaucugacuuugaagg	4	0	JT-2
guuaaugaucugacuuugaagg	6	0	JT-3
guuaaugaucugacuuugaagg	31	0	JCK-3
Auuaaugaucugacuuugaagg	1	1	LT-2
guuaaugauUugacuuugaagg	1	1	LT-1
guuaaugaucugacuuugaagA	1	1	LT-1
guuaaugaucugacuuugaagg	158	0	LCK-3
guuaaugaucugacuuugaagg	35	0	JT-1
guuaaugaucugacuuugaagg	72	0	LT-1
guAaaugaucugacuuugaagg	1	1	LT-2
guuaaugaucugacuuugaagg	23	0	JCK-2
guuaCugaucugacuuugaagg	1	1	LT-2
guuaaugaucugacuuugaagg	24	0	JCK-1
guuaauAaucugacuuugaaggc	1	1	LCK-3
guuaaugaucugacuuugaaggc	32	0	JT-1
Uuuaaugaucugacuuugaaggc	1	1	JT-1
guuaaugaucugacuuugaaggc	6	0	JT-2
guuaaugaucugacuuugaaggc	1	0	LCK-1
guuaaugaucugacuuugaaggc	59	0	LT-2
guuCaugaucugacuuugaaggc	1	1	JCK-3
guuaaugaucugacuuugaaggA	10	1	LT-2
Uuuaaugaucugacuuugaaggc	1	1	JCK-2
guuaaugaucugacuuugaaggc	56	0	JCK-2
guuaaugaucugacuuugaaggc	50	0	LT-3
guuaaugaucugacuuugaaggc	9	0	JT-3
guuaaugaucugacCuugaaggc	2	1	JCK-2
guuaaugaucugacuuugaaggA	8	1	JCK-2
guuaaugaucugacuuugaaggA	2	1	JCK-1
guuaaugaucugacuuugaaggA	2	1	JCK-3
guuaaugaucugacuuugaaggc	126	0	LCK-3
guuaaugaucugacuuugaaggc	57	0	JCK-1
gCuaaugaucugacuuugaaggc	1	1	JCK-3
guuCaugaucugacuuugaaggc	1	1	LCK-3
guuaaugaucugacuuuNaaggc	1	1	JCK-3
Auuaaugaucugacuuugaaggc	1	1	LT-2
guuaaugaucugacuuugaaggA	5	1	LT-1
guuaaugaucugacuuugaaggc	39	0	LT-1
guuCaugaucugacuuugaaggc	3	1	LT-2
guuaaugaucugacuuugaaggc	55	0	JCK-3
guuaaugaucugacuuugaaggcA	1	1	JT-2
guuaaugaucugacuuugaaggcu	32	0	LT-2
guuaaugaucugacuuugaaggcu	3	0	JT-2
guuaaugaucugacuuugaaggcu	18	0	LCK-3
guuaaugaucugacuuugaaggcu	13	0	JCK-1
guuaaugaucugacuuugaaggcu	11	0	LT-1
guuaaugaucugacuuugaaggcu	15	0	JCK-3
guuaaugaucugacuuugaaggcu	10	0	LT-3
guuaaugaucugacuuugaaggcA	1	1	JCK-1
guuaaugaucuNacuuugaaggcu	1	1	JCK-1
guuaaugaucugacuuugaaggcu	5	0	JT-3
guuaaugaucugacuuugaaggcu	16	0	JCK-2

```
                                                                                                                                                                   Star                                     Mature
uucgcgcagccccuguccaauucuaguuucaauuuuuauugugucacaauuaauucgaacggaacaacguggcaacuguucaauucugauuucaauuuuuuuuguuacauaauuaguucaaauaaaacaauuugacuacuguuuucuguuguagauuucuuagucauguuaaucuuuuuggcuuauucuuccagugaucgaccauguuaaugaucugacuuugaaggcuauuuagggcacuuaaauauau
.............................................................................................................................................................................................................guuaaugaucugacuuugaaggcua....................    31    0    LCK-3
.............................................................................................................................................................................................................guuaaugaucugacuuugaaggcua....................    14    0    LT-3
.............................................................................................................................................................................................................guuaaugaucugacuuugaaggcua....................    4     0    JCK-3
.............................................................................................................................................................................................................guuaaugaucugacuuugaaggcua....................    5     0    JCK-1
.............................................................................................................................................................................................................Auuaaugaucugacuuugaaggcua....................    1     1    JCK-3
.............................................................................................................................................................................................................guuaaugaucugacuuugaaggcua....................    18    0    LT-2
.............................................................................................................................................................................................................gNuaaugaucugacuuugaaggcua....................    1     1    JCK-1
.............................................................................................................................................................................................................guuaaugaucugacuuugaaggcua....................    14    0    LT-1
.............................................................................................................................................................................................................guuaaugaucugacuuugaaggcua....................    5     0    JCK-2
.............................................................................................................................................................................................................guuaaugaucugCcuuugaaggcua....................    1     1    LT-2
.............................................................................................................................................................................................................guuaaugaucugacuuugaaggcuau...................    2     0    LT-1
.............................................................................................................................................................................................................guuaaugaucugacuuugaaggcuau...................    4     0    LCK-3
.............................................................................................................................................................................................................guuaaugaucugacuuugaaggcuau...................    1     0    LT-3
.............................................................................................................................................................................................................guuaaugaucugacuuugaaggcuau...................    5     0    LT-2
.............................................................................................................................................................................................................guuaaugaucugacuuugaaggcuauu..................    1     0    LT-3
.............................................................................................................................................................................................................guuaaugaucugacuuugaaggcuauu..................    3     0    JCK-2
.............................................................................................................................................................................................................guuaaugaucugacuuugaaggcuauu..................    7     0    LCK-1
.............................................................................................................................................................................................................guuaaugaucugacuuugaaggcuauu..................    5     0    LCK-3
.............................................................................................................................................................................................................guuaaugaucugacuuugaaggcuauu..................    1     0    JCK-1
.............................................................................................................................................................................................................guuaaugaucugacuuugaaggcuauu..................    1     0    LT-1
.............................................................................................................................................................................................................guuaaugaucugacuuugaaggcuauu..................    10    0    LCK-2
.............................................................................................................................................................................................................guuaaugaucugacuuugaaggcuauu..................    2     0    LT-2
..............................................................................................................................................................................................................uuaaugaucugacuuugaag........................    8     0    LCK-3
..............................................................................................................................................................................................................uuaaugaucugacuuugaagg.......................    11    0    LT-1
..............................................................................................................................................................................................................uuaaugaucugacuuugaagg.......................    4     0    JCK-3
..............................................................................................................................................................................................................uuaaugaucugacuuugaagg.......................    3     0    JCK-2
..............................................................................................................................................................................................................uuaaugaucugacuuugaagg.......................    16    0    LT-3
..............................................................................................................................................................................................................uuaaugaucugacuuugaagg.......................    30    0    LCK-2
..............................................................................................................................................................................................................uuaaugaucugacuuugaagg.......................    27    0    LT-2
..............................................................................................................................................................................................................uuaaugaucugacuuugaagg.......................    22    0    LCK-1
..............................................................................................................................................................................................................uuaaugaucugacuuugaagg.......................    37    0    LCK-3
..............................................................................................................................................................................................................uuaaugaucugacuuugaagg.......................    1     0    JCK-1
..............................................................................................................................................................................................................uuaaugaucugacuuugaaggA......................    12    1    LCK-3
..............................................................................................................................................................................................................uuaaugaucugacuuugaaggc......................    12    0    LCK-3
..............................................................................................................................................................................................................uuaaugaucugacuuugaaggc......................    7     0    LT-3
..............................................................................................................................................................................................................uuaaugaucugacuuugaaggc......................    8     0    JCK-3
..............................................................................................................................................................................................................uuaaugaucugacuuugaaggc......................    4     0    LT-1
..............................................................................................................................................................................................................uuaaugaucugacuuugaaggc......................    10    0    JCK-2
..............................................................................................................................................................................................................uuaaugaucugacuuugaaggc......................    11    0    JT-1
..............................................................................................................................................................................................................uuaaugaCcugacuuugaaggc......................    1     1    JCK-1
..............................................................................................................................................................................................................uuaaugaucugacuuugaaggc......................    13    0    JCK-1
..............................................................................................................................................................................................................uuaaugaucugacuuugaaggA......................    1     1    LT-1
..............................................................................................................................................................................................................uuaaugaucugacuuugaaggc......................    14    0    LT-2
..............................................................................................................................................................................................................uuaaugaucugacuuugaaggcu.....................    9     0    LCK-3
..............................................................................................................................................................................................................uuaaugaucugacuuugaaggcu.....................    15    0    LT-2
..............................................................................................................................................................................................................uuaCugaucugacuuugaaggcu.....................    1     1    LT-2
..............................................................................................................................................................................................................uuaaugaucugacuuugaaggcu.....................    2     0    JCK-3
..............................................................................................................................................................................................................uuaaugaucugacuuugaaggcu.....................    4     0    LT-3
..............................................................................................................................................................................................................uuaaugaucugacuuugaaggcu.....................    4     0    JCK-1
..............................................................................................................................................................................................................uuaaugaucugacuuugaaggcu.....................    2     0    JCK-2
..............................................................................................................................................................................................................uuaaugaucugacuuugaaggcu.....................    1     0    LT-1
..............................................................................................................................................................................................................uuaaugaucugacuuugaaggcua....................    3     0    LT-3
..............................................................................................................................................................................................................uuaaugaucugacuuugaaggcua....................    2     0    LT-1
..............................................................................................................................................................................................................uuaaugaucugacuuugaaggcuau...................    32    0    LCK-1
..............................................................................................................................................................................................................uuaaugaucugacuuugaaggcuau...................    30    0    LCK-2
..............................................................................................................................................................................................................uuaaugaucugacuuugaaggcuau...................    1     0    LT-2
..............................................................................................................................................................................................................uuCaugaucugacuuugaaggcuau...................    1     1    LT-2
..............................................................................................................................................................................................................uuaaugaucugacuuugaaggcuauu..................    1     0    LT-1
...............................................................................................................................................................................................................uaaugaucugacuuugaa.........................    1     0    LT-3
...............................................................................................................................................................................................................uaaugaucugacuuugaag........................    4     0    LCK-3
...............................................................................................................................................................................................................uaaugaucugacuuugaagg.......................    5     0    LT-3
...............................................................................................................................................................................................................uaaugaucugacuuugaagg.......................    11    0    LCK-3
...............................................................................................................................................................................................................uaaugaucugacuuugaagg.......................    17    0    LCK-2
...............................................................................................................................................................................................................uaaugaucugacuuugaagg.......................    30    0    LCK-1
...............................................................................................................................................................................................................uaaugaucugacuuugaagg.......................    7     0    LT-2
...............................................................................................................................................................................................................uaaugaucugacuuugaagg.......................    2     0    JCK-3
...............................................................................................................................................................................................................uaaugaucugacuuugaagg.......................    4     0    LT-1
...............................................................................................................................................................................................................uaaugaucugacuuugaagg.......................    2     0    JCK-2
...............................................................................................................................................................................................................uaaugaucugacuuugaaggc......................    5     0    LT-1
...............................................................................................................................................................................................................uaaugaucugacuuugaaggA......................    3     1    LT-2
```

```
Star                                                                                                                                                                                          Mature
uucgcgcagccccuguccaauucuaguuucaauuuuuauuugugucacaauuaauucgaacggaacaacguggcaacuguucaauucugauuucaauuuuuuuuguuacauaauuaguucaaauaaaacaauuugacuacuguuuucuguuguagauuucuuagucauguuaaucuuuuuggcuuauucuuccagugaucgaccauguuaaugaucugacuuugaaggcuauuuagggcacuuaaauauau
................................................................................................................................................................................................................uaaugaucugacuuugaaggc......................    11    0    LT-2
................................................................................................................................................................................................................uaaugaucugacuuugaaggc......................    4    0    LT-3
................................................................................................................................................................................................................uaCugaucugacuuugaaggc......................    1    1    LT-2
................................................................................................................................................................................................................uaaugaucugaNuuugaaggc......................    1    1    JT-1
................................................................................................................................................................................................................uaaugaucugacuuugaaggc......................    6    0    JT-1
................................................................................................................................................................................................................uaaugaucugacuuugaaggc......................    7    0    LCK-3
................................................................................................................................................................................................................uaaugaucugacuuugaaggA......................    2    1    LT-1
................................................................................................................................................................................................................uaaugaucugacuuugaaggcA.....................    1    1    LT-3
................................................................................................................................................................................................................uaaugaucugacuuugaaggcu.....................    5    0    LT-2
................................................................................................................................................................................................................uaaugaucugacuuugaaggcua....................    3    0    LT-2
................................................................................................................................................................................................................uaaugaucugacuuugaaggcua....................    2    0    LT-1
.................................................................................................................................................................................................................aaugaucugacuuugaagg.......................    3    0    LT-1
.................................................................................................................................................................................................................aaugaucugacuuugaagg.......................    1    0    LCK-3
.................................................................................................................................................................................................................aaugaucugacuuugaagg.......................    2    0    LT-3
.................................................................................................................................................................................................................aaugaucugacuuugaaggc......................    1    0    LT-1
.................................................................................................................................................................................................................aaugaucugacuuugaaggc......................    2    0    LT-3
.................................................................................................................................................................................................................aaugaucugacuuugaaggcua....................    1    0    JCK-2
.................................................................................................................................................................................................................aaugaucugacuuugaaggcuauuuagg..............    2    0    LT-1
.................................................................................................................................................................................................................aaugaucugacuuugaaggcuauuuagg..............    2    0    LT-2
.................................................................................................................................................................................................................aaugaucugacuuugaaggcuauuuagggc............    1    0    LT-2
..................................................................................................................................................................................................................augaucugacuuugaagg.......................    2    0    LCK-3
..................................................................................................................................................................................................................augaucugacuNugaagg.......................    1    1    JT-1
..................................................................................................................................................................................................................augaucugacuuugaagg.......................    2    0    LT-3
..................................................................................................................................................................................................................augaucugacuuugaagg.......................    8    0    LT-2
..................................................................................................................................................................................................................augaucugacuuugaagg.......................    7    0    JT-1
..................................................................................................................................................................................................................augaucugacuuugaaggc......................    7    0    LT-2
..................................................................................................................................................................................................................augaucugacuuugaaggU......................    1    1    LT-2
..................................................................................................................................................................................................................augaucugacuuugaaggc......................    4    0    JCK-1
..................................................................................................................................................................................................................augaucugacuuugaaggc......................    4    0    JCK-3
..................................................................................................................................................................................................................augaucugacuuugaaggc......................    5    0    JCK-2
..................................................................................................................................................................................................................augaucugacuuugaaggc......................    7    0    LCK-3
..................................................................................................................................................................................................................augaucugacuuugaaggc......................    1    0    LT-1
..................................................................................................................................................................................................................augaucugacuuugaaggcu.....................    3    0    LT-2
..................................................................................................................................................................................................................augaucugacuuugaaggcu.....................    13    0    LCK-3
..................................................................................................................................................................................................................augaucugacuuugaaggcu.....................    2    0    JCK-2
..................................................................................................................................................................................................................augaucugacuuugaaggcu.....................    1    0    LT-3
..................................................................................................................................................................................................................augaucugacuuugaaggcua....................    1    0    LT-1
..................................................................................................................................................................................................................augaucugacuuugaaggcuauuuag...............    2    0    LT-1
..................................................................................................................................................................................................................augaucugacuuugaaggcuauuuag...............    5    0    LT-2
..................................................................................................................................................................................................................augaucugacuuugaaggcuauuuagg..............    2    0    LT-2
..................................................................................................................................................................................................................augaucugacuuugaaggcuauuuagg..............    4    0    LCK-3
..................................................................................................................................................................................................................augaucugaAuuugaaggcuauuuagg..............    1    1    LT-1
..................................................................................................................................................................................................................augaucugacuuugaaggcuauuuagg..............    2    0    LT-1
..................................................................................................................................................................................................................augaucugacuuugaaggcuauuuagg..............    6    0    LT-3
..................................................................................................................................................................................................................augaucugacuuugaaggcuauuuaggg.............    1    0    LT-3
..................................................................................................................................................................................................................augaucugacuuugaaggcuauuuaggg.............    2    0    LCK-3
...................................................................................................................................................................................................................ugaucugacuuugaaggA......................    2    1    LT-3
....................................................................................................................................................................................................................gaucugacuuugaaggcu.....................    1    0    LT-3
.....................................................................................................................................................................................................................aucugacuuugaaggcua....................    3    0    LCK-3
.....................................................................................................................................................................................................................aucugacuuugaaggcua....................    2    0    LT-3
.....................................................................................................................................................................................................................aucugacuuugaaggcuau...................    1    0    LT-1
.......................................................................................................................................................................................................................cugacuuugaCggcuauuuaggg.............    1    1    LT-1
........................................................................................................................................................................................................................ugacuuugaaggcuauuuagg..............    1    0    LCK-3
```
